# Hsp90 interacts with multiple dengue virus 2 proteins

**DOI:** 10.1038/s41598-018-22639-5

**Published:** 2018-03-09

**Authors:** Kanjana Srisutthisamphan, Krit Jirakanwisal, Suwipa Ramphan, Natthida Tongluan, Atichat Kuadkitkan, Duncan R. Smith

**Affiliations:** Institute of Molecular Biosciences, Mahidol University, Bangkok, Thailand

## Abstract

Infections with the mosquito-borne dengue virus (DENV) remain a significant public health challenge. In the absence of a commercial therapeutic to treat DENV infection, a greater understanding of the processes of cellular replication is required. The abundant cellular chaperone protein heat shock protein 90 (Hsp90) has been shown to play a proviral role in the replication cycle of several viruses, predominantly through the stabilization of specific viral proteins. To investigate any potential role of Hsp90 in DENV infection the interaction between Hsp90 and DENV proteins was determined through co-immunoprecipitation experiments. Six DENV proteins namely envelope (E) and nonstructural (NS) proteins NS1, NS2B, NS3, NS4B and NS5 were shown to interact with Hsp90, and four of these proteins (E, NS1, NS3 and NS5) were shown to colocalize to a variable extent with Hsp90. Despite the extensive interactions between Hsp90 and DENV proteins, inhibition of the activity of Hsp90 had a relatively minor effect on DENV replication, with inhibition of Hsp90 resulting in a decrease of cellular E protein (but not nonstructural proteins) coupled with an increase of E protein in the medium and an increased virus titer. Collectively these results indicate that Hsp90 has a slight anti-viral effect in DENV infection.

## Introduction

Dengue virus (DENV) is an arthropod-borne virus that can cause a febrile illness (dengue fever, DF) in humans that is sometimes associated with severe bleeding (dengue hemorrhagic fever, DHF) and hypovolemic shock (dengue shock syndrome, DSS)^1^. DENV is transmitted to humans by the bite of an infected *Aedes* mosquito (*Aedes aegypti, Aedes albopictus*)^2^. The first reported DENV epidemic is believed to have occurred in 1779, but it was after World War II that a more severe disease presentation emerged^3^. DENV transmission has been reported in about 100 tropical and sub-tropical countries around the world, and it is currently estimated that 400 million DENV infections occur each year, with nearly 100 million of those cases having symptomatic presentation to some degree^4^. DENV is an enveloped positive-sense single-stranded RNA virus that is a member of *Flaviviridae* family, genus *Flavivirus* and species *Dengue virus*, with four distinct serological types (DENV 1, 2, 3 and 4). The RNA genome of DENV is approximately 11 Kb and encodes for three structural proteins which are the envelope glycoprotein (E), membrane (M) and capsid (C) proteins and seven nonstructural proteins (NS) designated NS1, NS2A, NS2B, NS3, NS4A, NS4B and NS5^5^.

Cellular infection with DENV starts with the attachment of the virus to host cell surface proteins and a number of putative receptors have been identified (reviewed in^6,7^). Several of these putative receptor proteins including GRP78 (a member of the heat shock 70 family) and heat shock proteins 70 and 90 (Hsp70 and Hsp90) are classed as chaperone proteins^8^, and it is likely that there are interactions between the identified chaperone proteins and viral proteins during infection, in addition to the initial receptor interaction^9^. For example while GRP78 has been identified as a receptor protein for DENV^10^, cellular interactions between DENV proteins and GRP78 have also been reported^11,12^. Similarly Hsp70 and Hsp90 have been identified as receptor proteins for DENV^5^, and different Hsp70 isoforms have been shown to be critical mediators of several stages of the DENV replication cycle^13^.

Hsps are chaperones that play important roles in regulating the folding or unfolding of polypeptides, regulating transport and translocation of proteins into their correct subcellular compartment, and protein degradation^14^. Hsp90, with a molecular weight of 90 kDa, is a highly abundant and essential chaperone protein in prokaryotic and eukaryotic cells, with Hsp90 making up 1–2% of total cellular protein within unstressed cells and this can double under stress^15^. Hsp90 participates in multiple biological processes including cell cycle control, cell survival, intracellular transport, protein degradation, signal transduction, and the response to cellular stress^16^. Hsp90 also facilitates the final maturation folding of proteins such as p53 and steroid hormone receptors (SHRs) and the refolding of denatured proteins under stress^17^. In mammals, there are two cytoplasmic isoforms of Hsp90. Hsp90α is stress inducible, while Hsp90β is a constitutively expressed protein and the two proteins have 85%, sequence similarity^18^. Hsp90 forms a homodimer with each monomer composing of three domains (N-terminal, middle (M), and C-terminal) with a highly charged linker region connecting the N and M domains. The N-terminal (N domain) is composed of a co-chaperone binding motif and an ATP binding site^15^. The ATP/ADP binding region is a conserved region forming a lid which closes during ATP binding and opens during ADP binding^15^. The M domain is the major interaction site for client proteins, and is involved in ATP hydrolysis^19^. The C-terminus contains the dimerization motif and an alternative ATP/ADP and drug binding site. Moreover this domain has a conserved MEEVD motif that is a tetratricopeptide (TPR)-containing chaperone binding site, which is recognized by co-chaperones such as Hop (Hsp70-Hsp90 organizing protein)^20^ and cyclofilin-40^21^. Hsp90 activity requires a co-chaperone protein or a client protein^22^, and it is known that different combinations of co-chaperones associate with different conformational states of Hsp90^23^.

Previous reports have shown that Hsp90 has a role in the replication of a number of viruses, including mumps virus^24^, hepatitis C virus^25^, rabies virus^26^ and chikungunya virus^27,28^. In all of these studies the action of Hsp90 promotes virus replication, often through interacting with, and stabilizing specific viral proteins. While it is known that Hsp90 interacts with DENV E protein^5^, little is known about any interaction between Hsp90 and other DENV proteins. This study therefore sought to systematically survey all ten DENV proteins to determine if any proteins besides DENV E protein interacted with Hsp90, as well as more broadly investigate the role of Hsp90 in DENV replication.

## Results

### Expression of Hsp90 in DENV 2 infected cells

**Figure 1:**
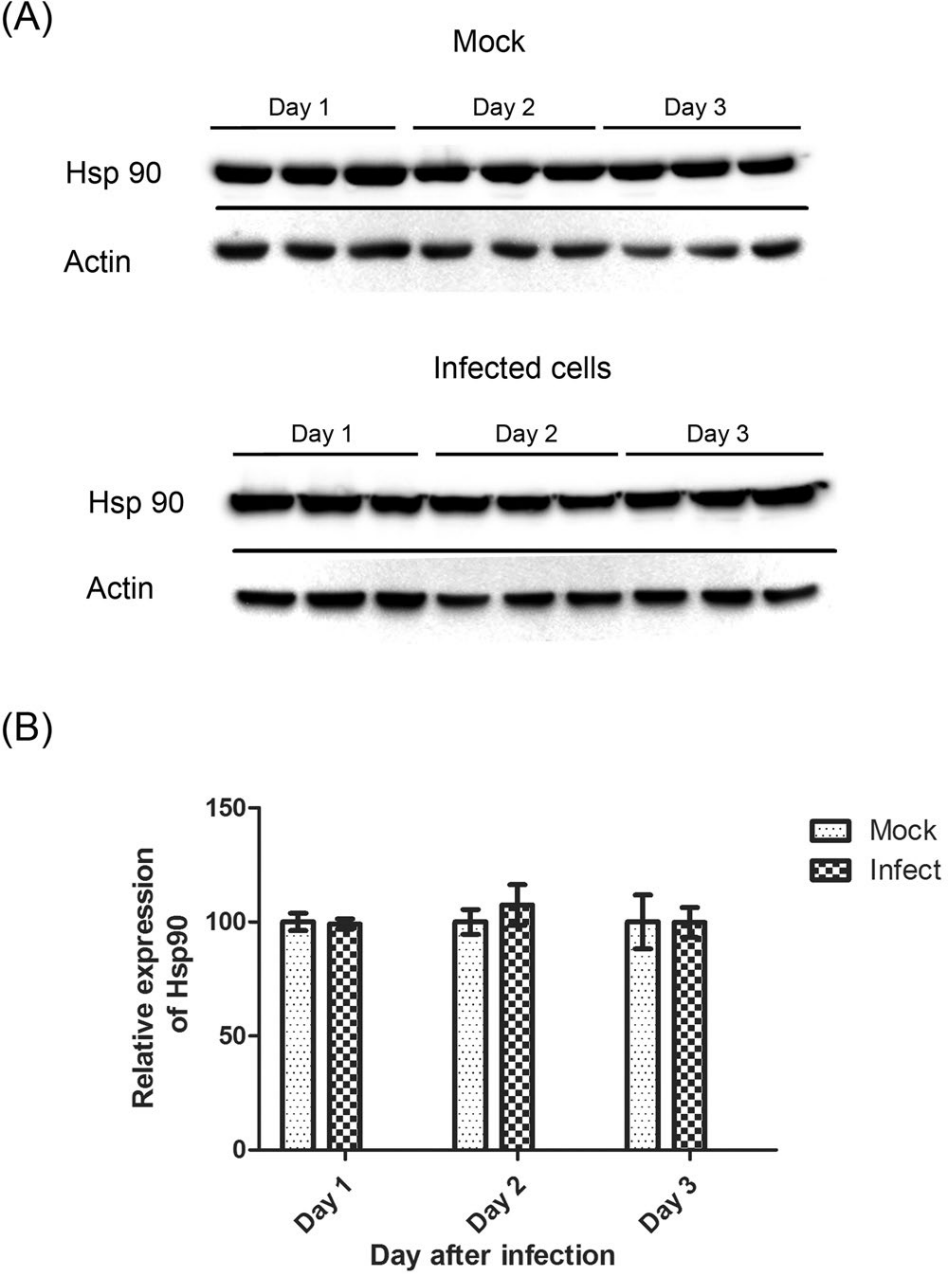
Hsp90 expression during DENV 2 infection. HEK293T/17 cells were infected with DENV 2 at MOI 1 or mock infected independently in triplicate, and cells were collected at days 1, 2 and 3 post- infection (p.i). Proteins were separated by SDS-PAGE and then transferred to a nitrocellulose membrane. (**A**) Membrane was probed with a polyclonal anti-Hsp90 antibody as primary antibody followed by an appropriate HRP-conjugated secondary antibody. Filter was re-probed with an anti-actin antibody. Black lines separate the different probing. (**B**) Band intensities from the western blot were quantitated by Quantity One software and are displayed graphically. Uncropped images are presented in the supplemental materials.

To determine the intracellular levels of Hsp90 during infection, HEK293T cells were infected with DENV 2 at MOI 1. On day 1, 2 and 3 p.i, total cellular proteins were extracted, separated by electrophoresis and transferred to nitrocellulose membranes before being subjected to western blot analysis to determine expression of Hsp90 with actin as a loading control. The results (Fig. 1A) showed the expected band sizes of 90 (Hsp90) and 43 kDa (actin). Image quantitation of signals from the three independent replicates (Fig. 1B) after normalization against actin showed that levels of Hsp90 were not significantly changed during DENV infection.

### The interaction of Hsp90 and DENV proteins

**Figure 2:**
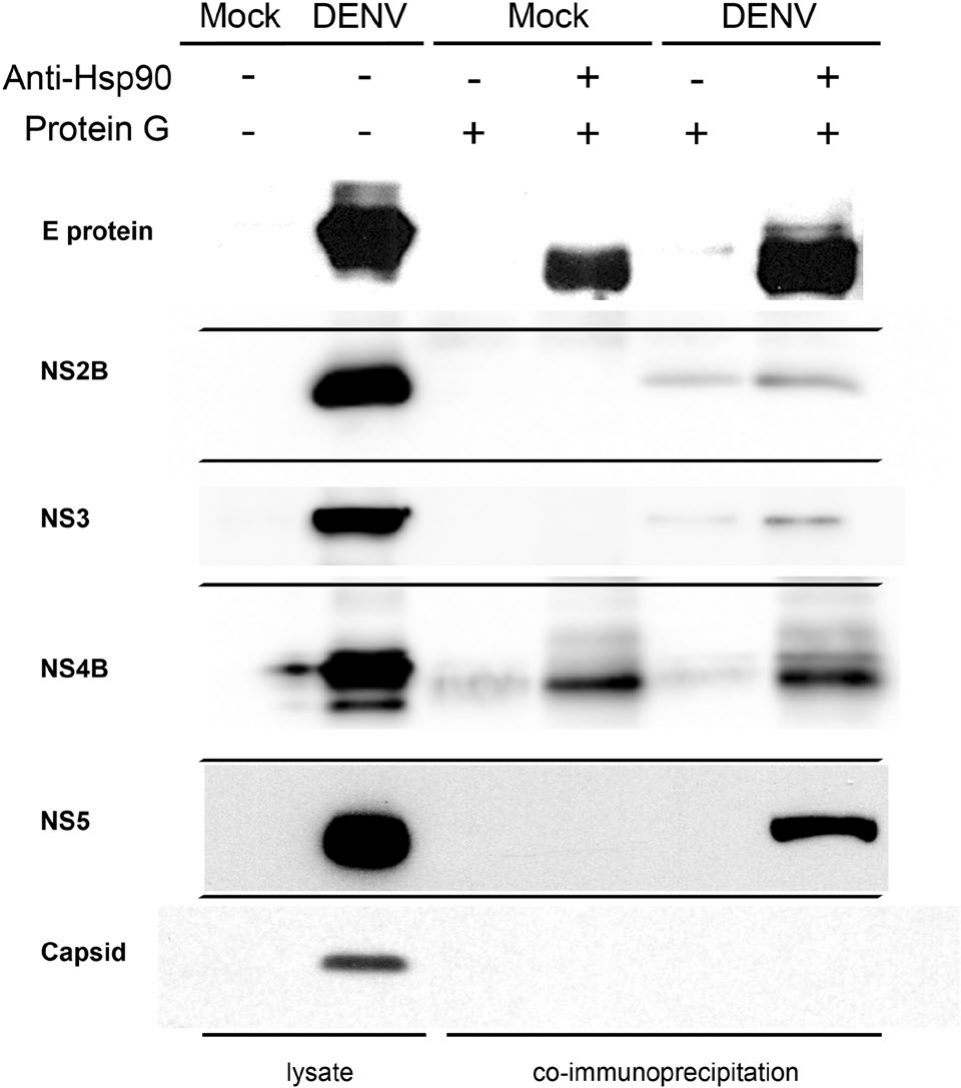
Hsp90 co-immunoprecipitation (co-IP) analysis of DENV proteins. HEK293T/17 cells were infected with DENV 2 at MOI 1 and then the cells were collected at day 2 p.i. Co-immunoprecipitation (co-IP) assay was performed using an anti-Hsp90α/β (H-114) antibody and membranes were screened by western blot analysis for co-immunoprecipitating DENV proteins. Proteins from lysates without pull-down confirmed the presence of the DENV proteins as appropriate. Different filters are separated by thin black lines. Uncropped images are presented in the supplemental materials.

To investigate the interaction between Hsp90 and DENV proteins, co-immunoprecipitation (Co-IP) assays were performed. We initially sourced 9 antibodies, directed against DENV capsid, prM, E, NS1, NS2B, NS3, NS4A, NS4B and NS5, but were unable to commercially source an antibody directed against NS2A. Three antibodies, prM, NS1 and NS4A showed no signal when evaluated in western analysis of lysates of DENV infected HEK293T/17 cells (data not shown). To evaluate the interaction of the remaining 6 proteins with Hsp90, HEK293T/17 cells were either mock infected or infected with DENV 2 and on day 2 p.i., total cellular proteins were extracted. After pre-clearing with protein G sepharose beads, the cell lysates were incubated with or without an anti-Hsp90 antibody followed by incubation with protein G sepharose beads. The co-immunoprecipitated protein complexes were eluted and analyzed by western blotting using antibodies against DENV E, capsid, NS2B, NS3, NS4B and NS5. Co-immunoprecipitating bands were seen for DENV E, NS2B, NS3, NS4B and NS5 (Fig. 2). While the antibody used could detect capsid protein in western analysis (Fig. 2) there was no evidence of co-immunoprecipitation with Hsp90.

**Figure 3:**
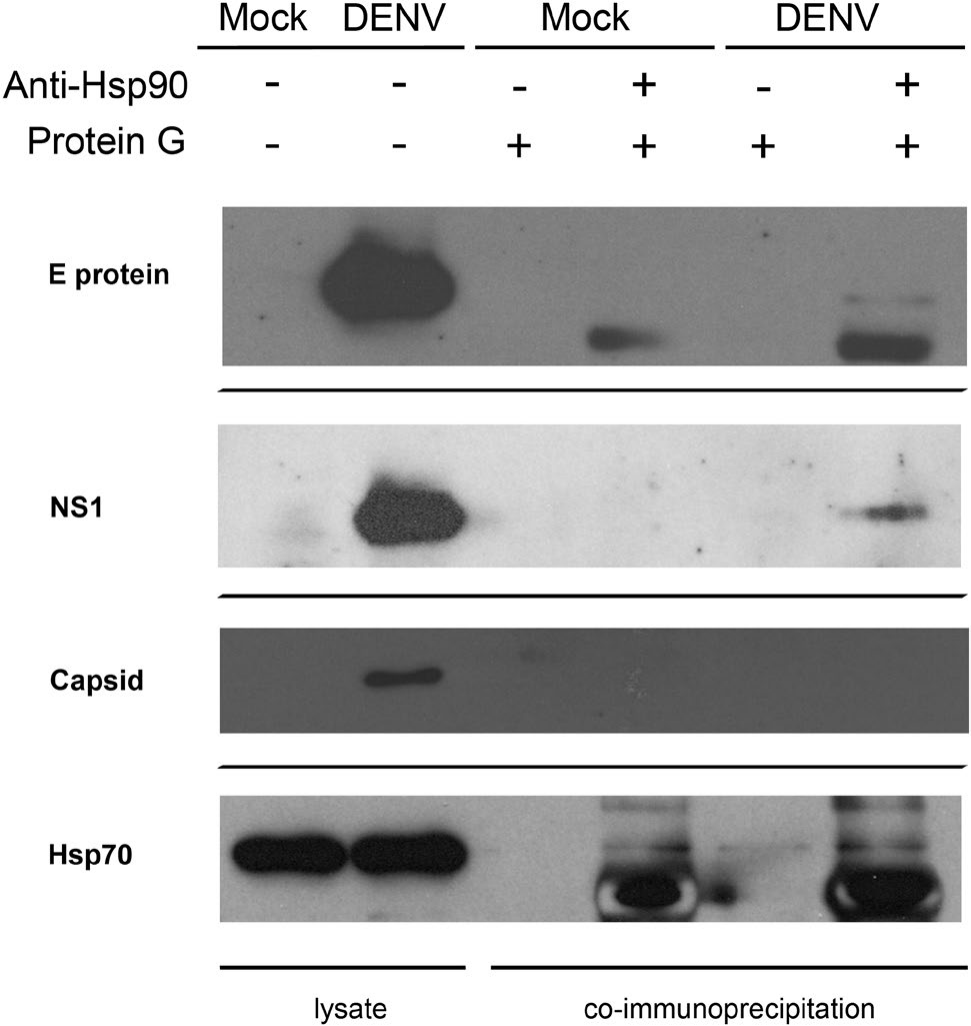
Hsp90 co-immunoprecipitation (co-IP) analysis of selected DENV proteins. HepG2 cells were infected with DENV 2 at MOI 20 and then the cells were collected at day 2 p.i. Co-immunoprecipitation (co-IP) assay was performed using an anti-Hsp90α/β (H-114) antibody and membranes were screened by western blot analysis for co-immunoprecipitating DENV proteins. One filter was probed with an antibody directed against Hsp70 as a co-immunoprecipitation control. Proteins from lysates without pull-down confirmed the presence of the DENV proteins or Hsp70 as appropriate. Different filters are separated by thin black lines. Uncropped images are presented in the supplemental materials.

The experiment was repeated for selected proteins, but this time with DENV infected HepG2 cells. After infection and immunoprecipitation the lysates were probed for interactions between E protein, NS1 and capsid protein. In addition the membrane was probed with a known Hsp90 interacting protein, namely Hsp70. Results (Fig. 3) showed interactions between Hsp90 and DENV E protein and NS1, but no signal was again observed for DENV capsid protein. The presence of Hsp70 in the immunoprecipitated complex (Fig. 3) confirms the lack of an interaction between DENV capsid protein and Hsp90.

**Figure 4:**
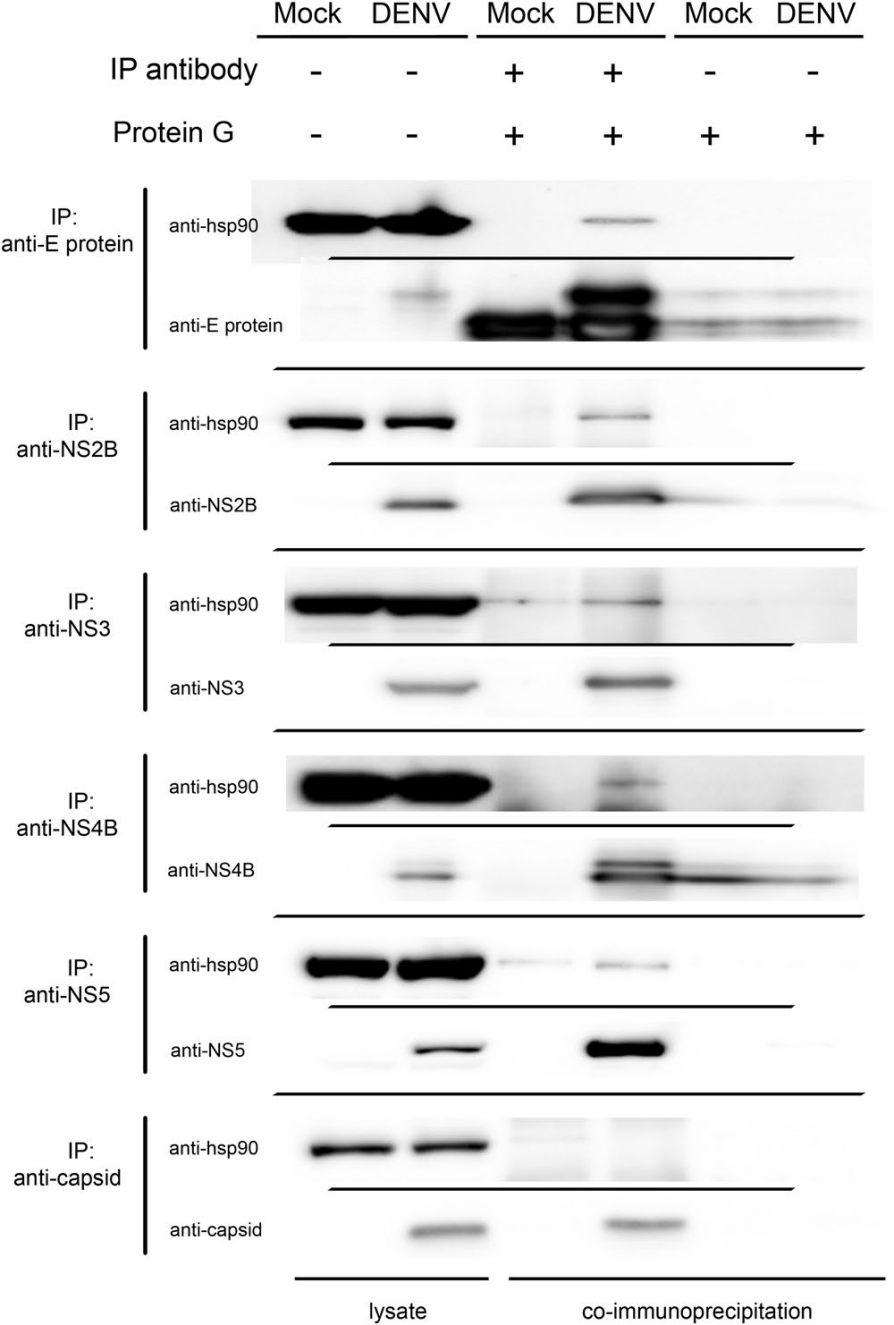
Western blot analysis of the interaction between Hsp90 and DENV proteins by reverse Co-IP assay. HEK293T/17 cells were infected with DENV 2 at MOI 1 and then the cells were collected at day 2 p.i. Reverse co-immunoprecipitation assays were performed using antibodies specific to DENV proteins to pull down immunoprecipitating complexes, followed by western blot analysis to detect Hsp90. The presence of Hsp90 protein in cell lysate and reverse co-IP products were detected by western blot analysis using an anti-Hsp90α/β antibody (H-114). Filters were stripped and re-probed with the anti-DENV antibody used in the original pull-down. Different filters/probing are separated by thin black lines. Uncropped images are presented in the supplemental materials.

To confirm the interaction between Hsp90 and DENV proteins, reverse co-immunoprecipitations were performed. HEK293T/17 cells were mock infected or infected with DENV 2. On day 2 p.i., the immunoprecipitations were repeated, but this time the immunoprecipitating antibody was directed against DENV E protein, capsid, NS2B, NS3, NS4B and NS5 as appropriate. Membranes were initially probed with an antibody directed against Hsp90, and subsequently with an antibody directed against the immunoprecipitating protein. Results (Fig. 4) confirmed the interaction between Hsp90 and DENV E, NS2B, NS3, NS4B and NS5 and again confirmed the lack of an interaction between Hsp90 and DENV capsid protein.

### The interaction between Hsp 90 and non-structural 3 or 5 proteins of dengue virus

**Figure 5:**
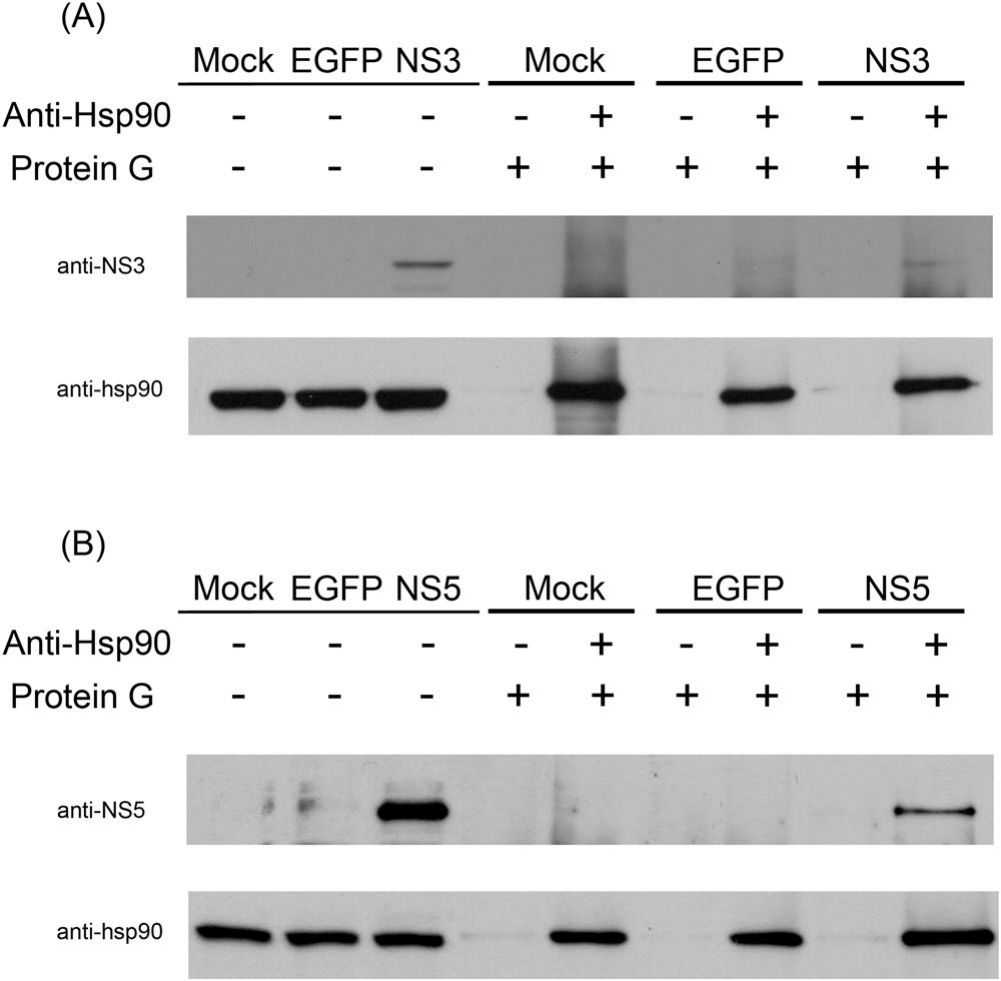
Analysis of the interactions between Hsp90 and DENV NS3 and NS5 proteins. HEK293T/17 cells were transfected with pCDNA_EGFP (control), pCDNA_D2NS3 or pEGFPC2_D2NS5 individually, and proteins were collected at day 2 post-transfection. Co-immunoprecipitation assays were performed using an anti-Hsp90α/β (H-114) and filters were subjected to western blot analysis to detect the presence of (**A**) DENV NS3 or (**B**) DENV NS5 proteins. The filters were subsequently probed with the anti-Hsp90 antibody used in the original pull down. Different probing are separated by white space. Uncropped images are presented in the supplemental materials.

The interaction between DENV NS3 and NS5 has been clearly established^29^. It is possible therefore that while both NS3 and NS5 co-immunoprecipitated with Hsp90, Hsp90 could be interacting with only one of the two proteins, with the second protein being co-immunoprecipitaed though its interaction with the other DENV protein. To address this issue DENV 2 full length NS3 and NS5 were cloned in mammalian expression vectors, with NS5 being cloned in frame with EGFP. These constructs were transfected into HEK293T/17cells, and the efficiency of transfection was determined by examination under a fluorescence microscope (for the NS5 construct) and by western blotting for both constructs. Results showed robust transfection levels for the NS5 construct as determined by fluorescent microscopy (Supplemental Figure S1A) and by western blotting for both constructs (Supplemental Figure S1B). HEK2973T/17 cells were therefore transfected separately with both constructs, together with an EGFP construct control, and subjected to immunoprecipitation with an antibody directed against Hsp90 with the western blots being probed with antibodies against NS3 or NS5 as appropriate. After the first western blot, the filters were stripped and re-probed with an antibody against Hsp90. Results (Fig. 5) clearly demonstrate that both NS3 and NS5 interacted independently with Hsp90.

### Colocalization between Hsp90 protein and DENV proteins

**Figure 6:**
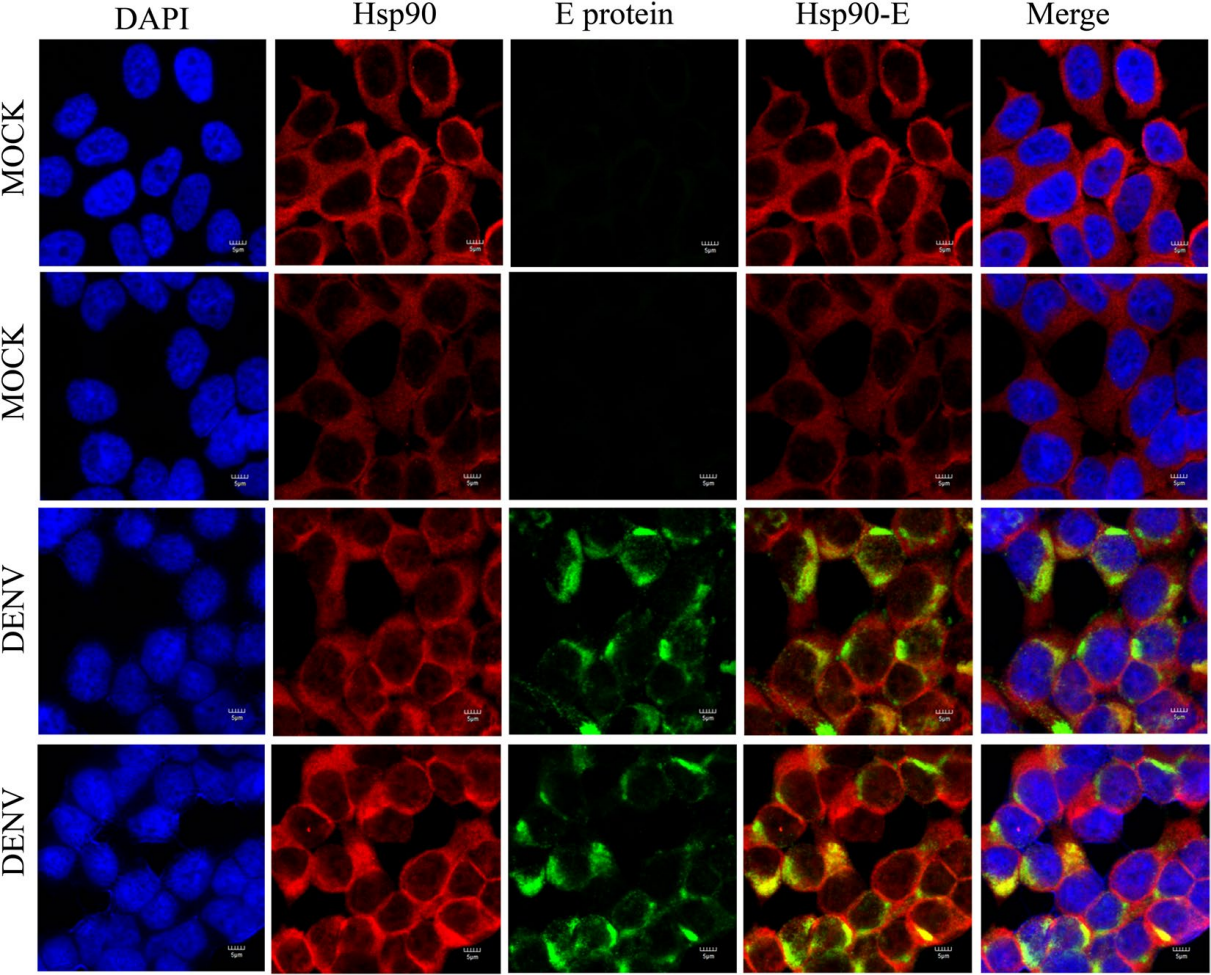
Colocalization of Hsp90 and DENV envelope (E) protein. HEK293T/17 cells were grown on cover slips and then infected with DENV 2 at MOI 1 or mock infected. At 48 hr p.i the cells were fixed, permeabilized and incubated primary antibodies directed against DENV E protein (green) and Hsp90 (red), followed by incubation with appropriate secondary antibodies. Cells were subsequently stained with DAPI (blue). Images were collected on an Olympus FluoView 1000 confocal microscope with magnification 180X.

**Figure 7:**
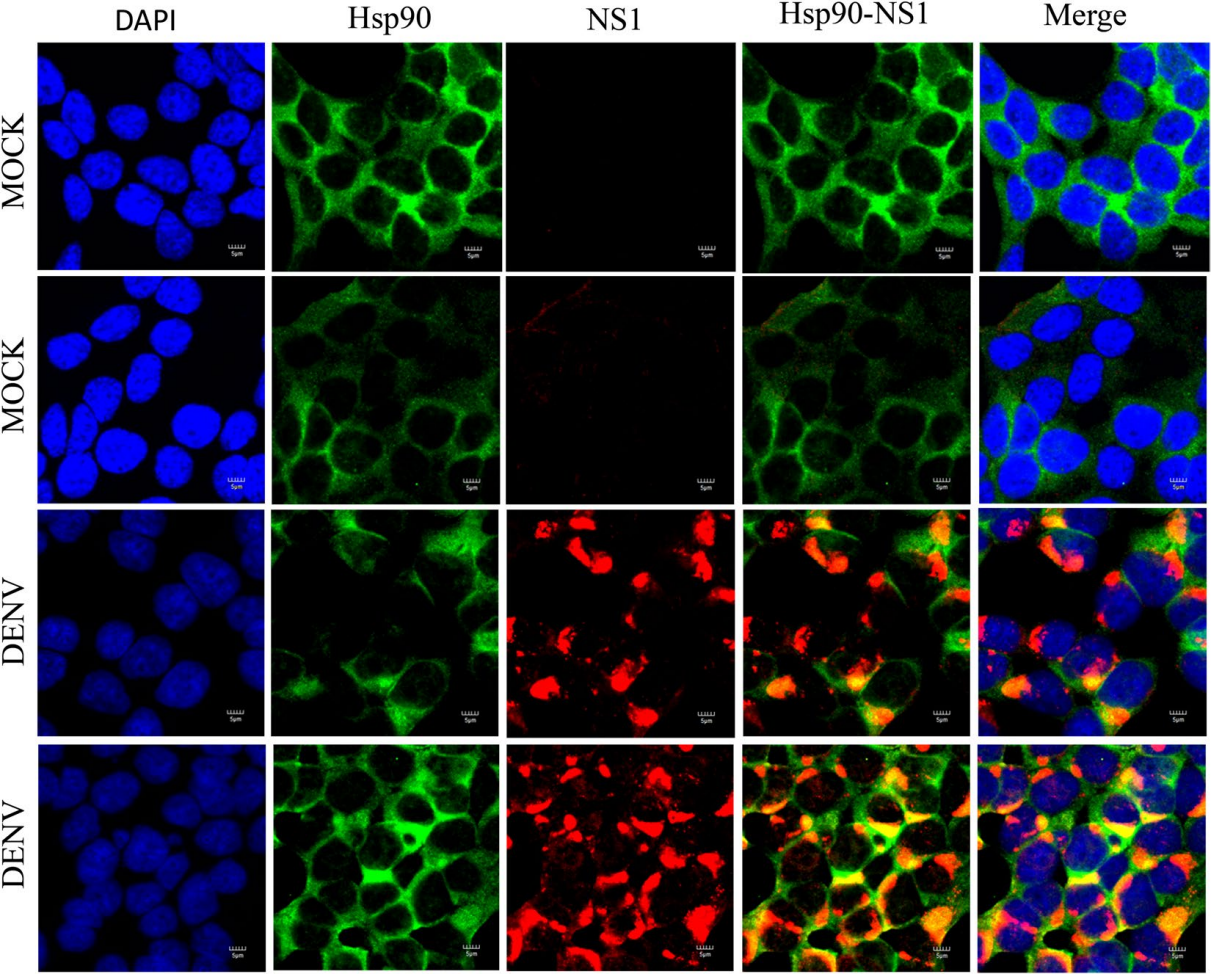
Colocalization of Hsp90 and DENV NS1 protein. HEK293T/17 cells were grown on cover slips and then infected with DENV 2 at MOI 1 or mock infected. At 48 hr p.i the cells were fixed, permeabilized and incubated primary antibodies directed against DENV NS1 protein (red) and Hsp90 (green), followed by incubation with appropriate secondary antibodies. Cells were subsequently stained with DAPI (blue). Images were collected on an Olympus FluoView 1000 confocal microscope with magnification 180X.

**Figure 8:**
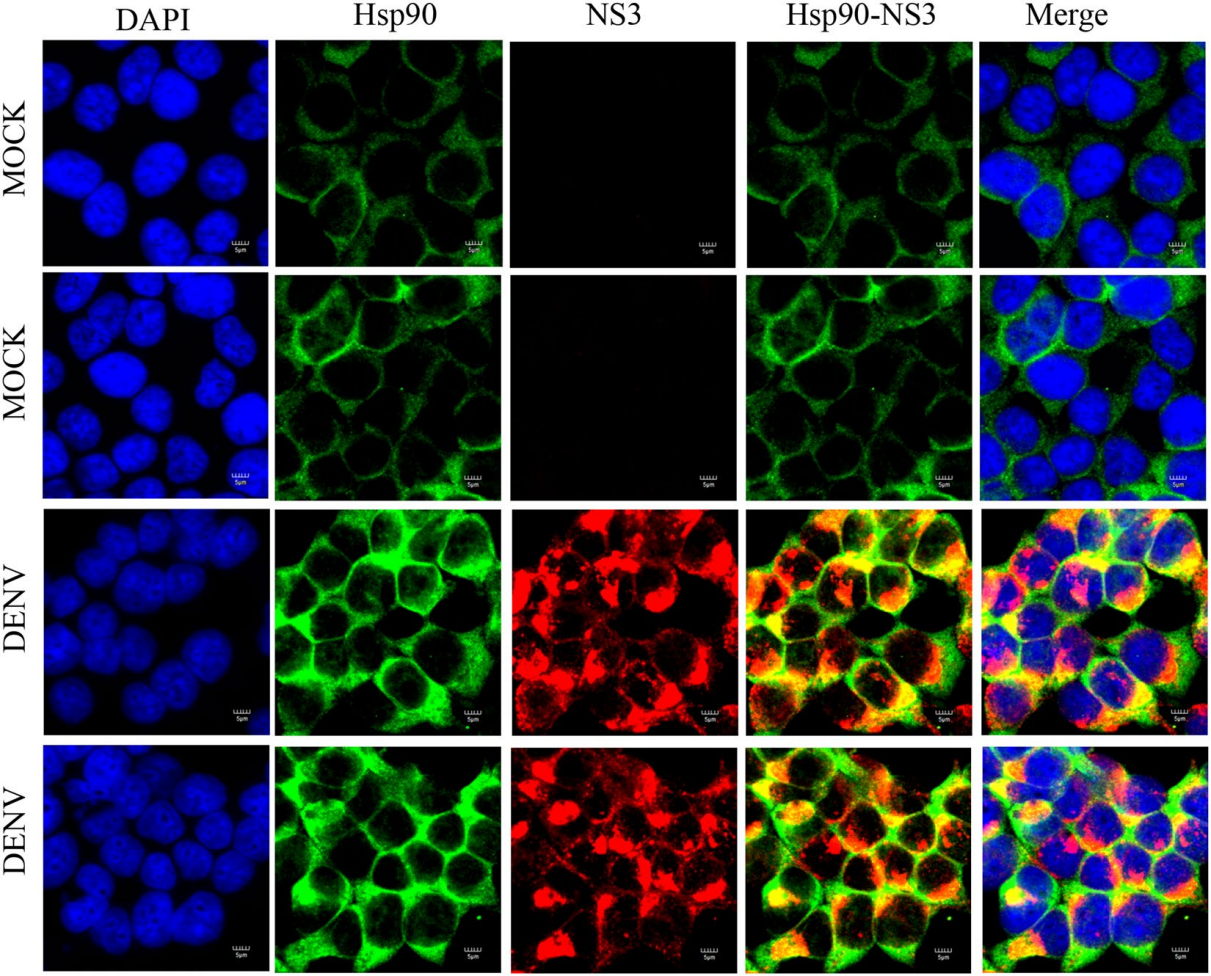
Colocalization of Hsp90 and DENV NS1 protein. HEK293T/17 cells were grown on cover slips and then infected with DENV 2 at MOI 1 or mock infected. At 48 hr p.i the cells were fixed, permeabilized and incubated primary antibodies directed against DENV NS1 protein (red) and Hsp90 (green), followed by incubation with appropriate secondary antibodies. Cells were subsequently stained with DAPI (blue). Images were collected on an Olympus FluoView 1000 confocal microscope with magnification 180X.

**Figure 9:**
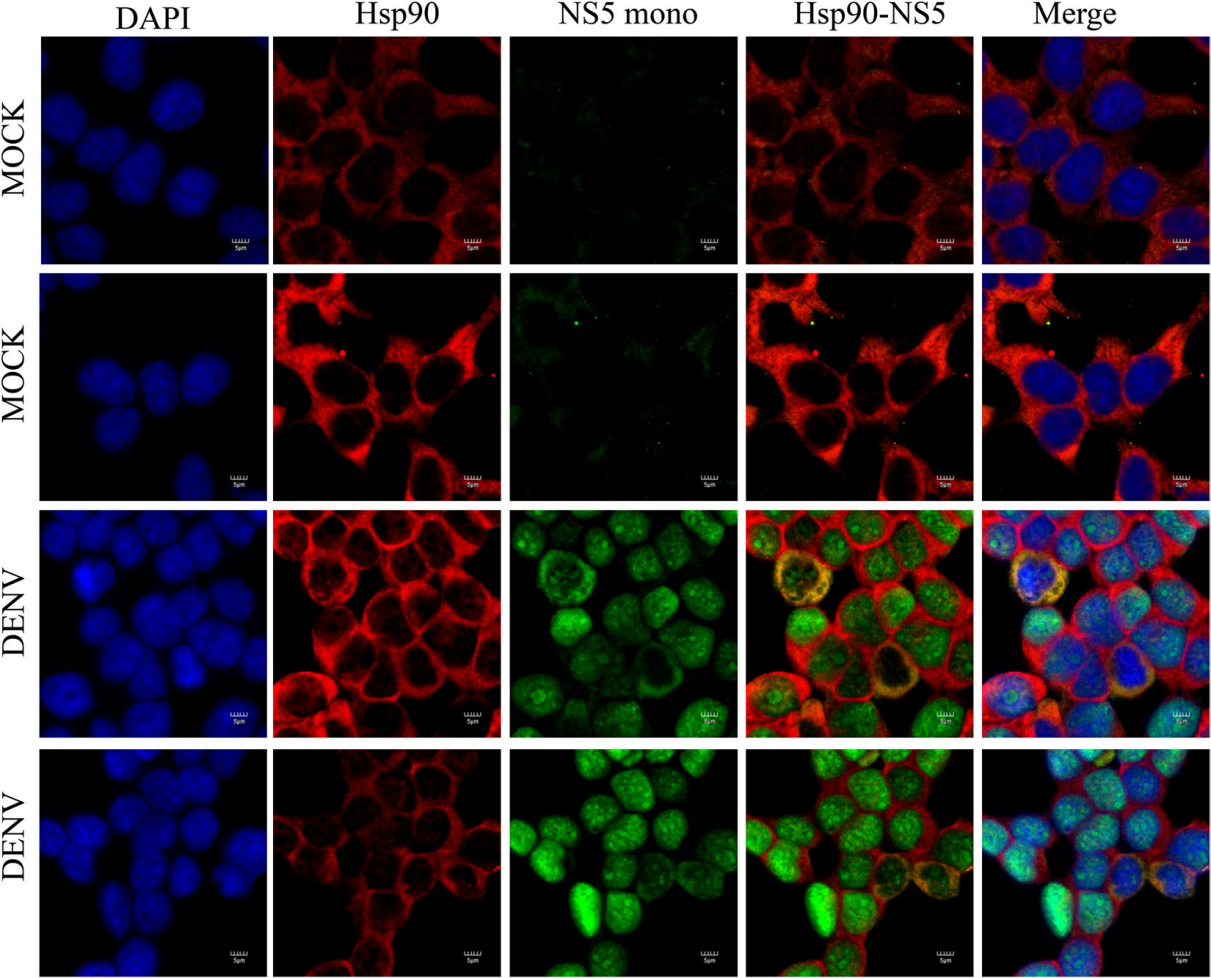
Colocalization of Hsp90 and DENV NS5 protein. HEK293T/17 cells were grown on cover slips and then infected with DENV 2 at MOI 1 or mock infected. At 48 hr p.i the cells were fixed, permeabilized and incubated with a polyclonal anti-Hsp90 antibody (red) and a monoclonal anti-DENV NS5 (green) antibody followed by incubation with appropriate secondary antibodies. Cells were subsequently stained with DAPI (blue). Images were collected on an Olympus FluoView 1000 confocal microscope with magnification 180X.

**Figure 10:**
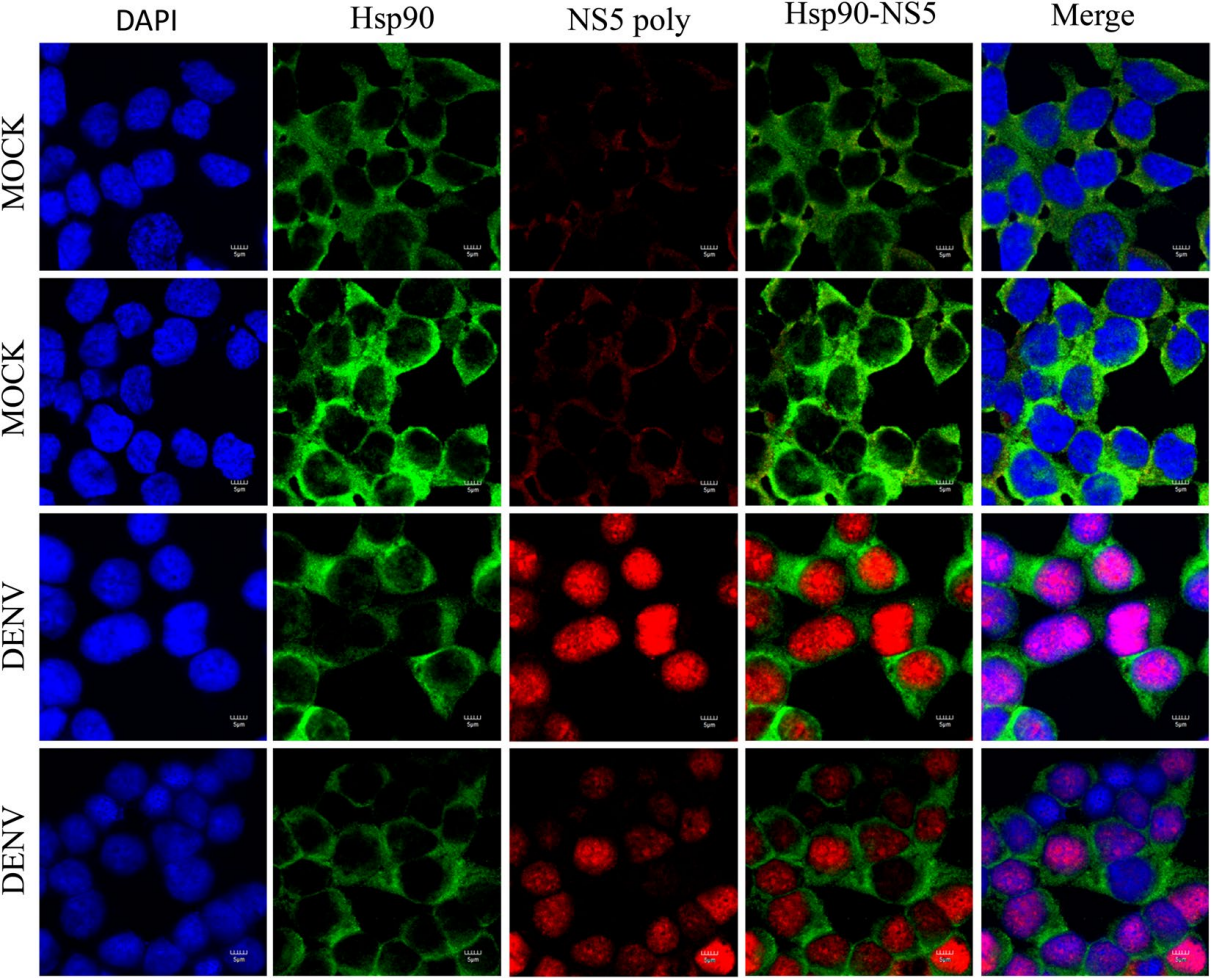
Colocalization of Hsp90 and DENV NS5 protein. HEK293T/17 cells were grown on cover slips and then infected with DENV 2 at MOI 1 or mock infected. At 48 hr p.i the cells were fixed, permeabilized and incubated with a polyclonal anti-Hsp90 antibody (green) and a polyclonal anti-DENV NS5 (red) antibody followed by incubation with appropriate secondary antibodies. Cells were subsequently stained with DAPI (blue). Images were collected on an Olympus FluoView 1000 confocal microscope with magnification 180X.

To determine if Hsp90 was colocalizing with the DENV proteins, HEK293T/17 cells were mock infected or infected with DENV 2, and colocalization between Hsp90 and DENV E, NS1, NS3 and NS5 proteins was examined by immunofluorescence on day 2 p.i. using a confocal microscope. Results (Figs 6–10) showed some colocalization between Hsp90 and all of the proteins examined, albeit to different degrees. DENV E protein showed some colocalization (Fig. 6), while NS1 (Fig. 7) and NS3 (Fig. 8) showed considerable levels of colocalization. NS5 expression was observed with two different antibodies, a monoclonal antibody (Fig. 9) and a polyclonal antibody (Fig. 10). The monoclonal antibody showed some cytoplasmic colocalization between Hsp90 and NS5 (Fig. 9), while the polyclonal antibody which only detected NS5 present in the nucleus, showed no colocalization with Hsp90 (Fig. 10).

### Effect of inhibition of Hsp90 activity on DENV infection

Geldanamycin (GA) is a well characterized inhibitor of Hsp90 with a minimal effective concentration of 300 nM (manufacturers’ recommendation). However GA has well characterized cytotoxicity. We therefore evaluated the cytotoxicity of GA towards HEK293T/17 cells, with evaluation at 24 and 48 h post treatment (h.p.t). The results (Supplemental Figure S2) showed that GA was significantly cytotoxic to cells at concentrations above 100 nM when assayed at 24 h.p.t, and significantly cytotoxic to cells at concentrations above 50 nM at 48 h.p.t (Supplemental Figure S2B). The morphology of cells was monitored by light microscopy. Morphological changes were observed with cells treated with concentrations above 500 nM (Supplemental Figure S3A,B) and cells treated with 300 nM GA were normal as assessed by gross morphology (Supplemental Fig. 3A,B). Levels of expression of Hsp90 were unaffected by geldanamycin treatment up to 800 nM (Supplemental Figure S4A,B). Therefore despite the observed cytotoxicity, the manufacturers recommended minimal effective (300 nM) was used in subsequent experiments.

**Figure 11:**
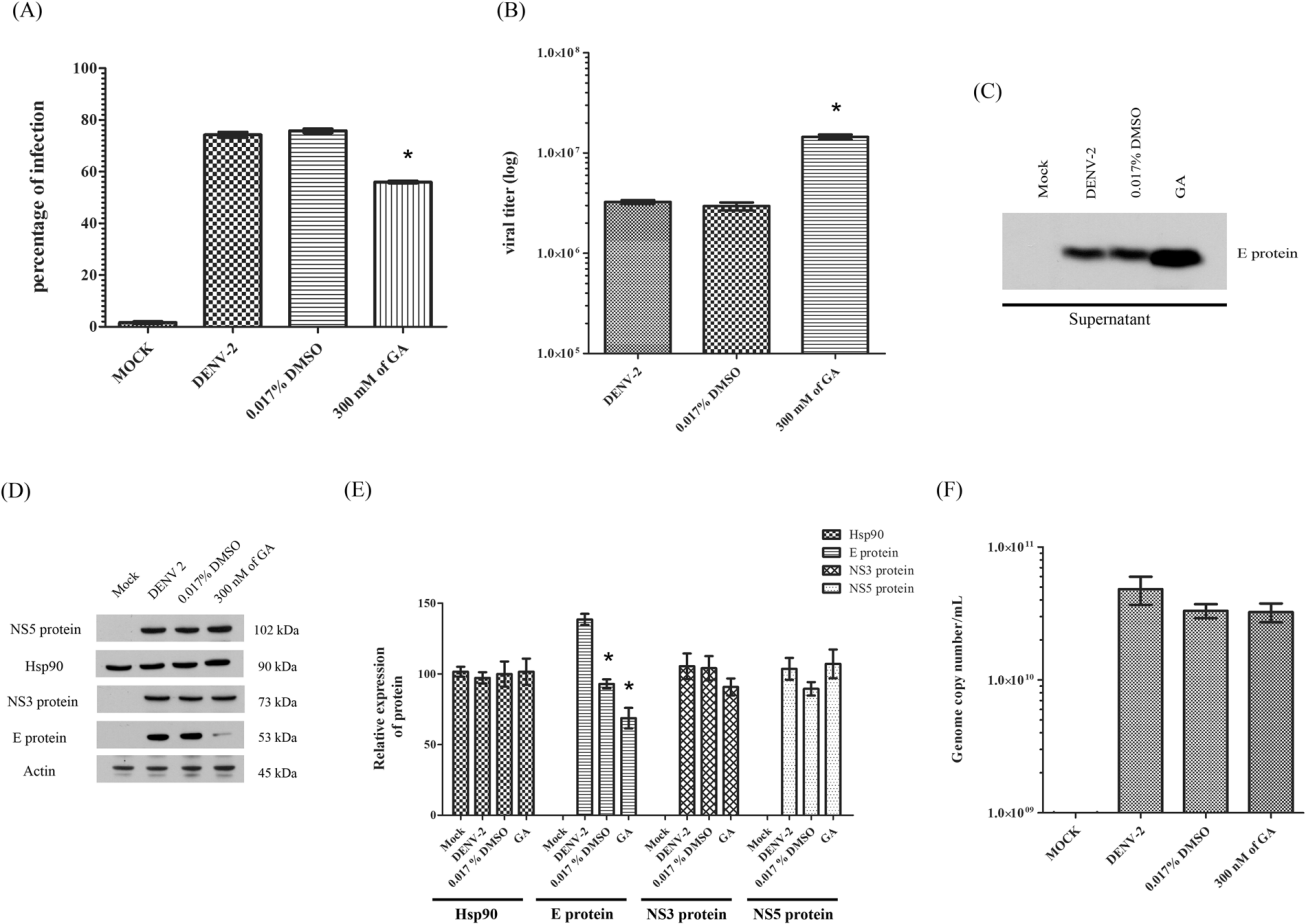
Effect of inhibition of Hsp90 on DENV infection. HEK293T/17 cells were treated with geldanamycin (GA) at a concentration of 300 nM followed by mock infection infection with DENV 2 at MOI 1. Controls included untreated mock infected cells, untreated DENV infected cells and vehicle (0.017% DMSO) treated cells. Cells were collected at 48 h.p.i. post treatment and (**A**) the percentage of infections determined by flow cytometry; (**B**) the viral titer determined plaque assay; (**C**) the level of E protein in the supernatant determined by western blot analysis; (**D**) the levels of DENV NS5, NS3 and E protein together with Hsp90 and actin determined by western blot analysis followed by (**E**) determination of band intensities by Quantity One software and (**F**) determination of DENV copy number by real-time quantitative PCR. All experiments were undertaken independently in triplicate, with duplicate plaque assay where appropriate. Data is plotted as mean ± S.D. *P-value < 0.05. Different western blots are separated by white space. Uncropped images are presented in the supplemental materials.

To determine the effect of inhibition of Hsp90 on DENV infection, HEK293T/17 cells were incubated with 300 nM of GA for 2 hr, followed by mock infection or infection with DENV 2 in the absence of GA, after which medium was removed and replaced with fresh medium containing GA. On day 2 p.i. the cells were harvested for flow cytometry and western blot analysis, while the supernatant was collected for plaque assay and western blot analysis. The result from flow cytometry showed that the percentage of infection was significantly decreased when the cells were treated with GA as compared with untreated cells, or cells treated with vehicle alone (Fig. 11A), while the plaque assay results showed increased levels of infectious virus in the supernatant in the GA treated samples as opposed to the control treated samples (Fig. 11B). The increased presence of the virus in the supernatant after GA treatment was confirmed by western blotting (Fig. 11C). The cellular expression levels of DENV E NS3 and NS5 proteins were determined by western blot together with examination of the levels of Hsp90 and actin as a control. Results (Fig. 11D) showed that there was no change in the levels of DENV NS3 or NS5 proteins, and levels of Hsp90 were unaltered by treatment with GA. However, clearly reduced levels of DENV E protein were observed after GA treatment (Fig. 11D,E). Finally the effect of GA on the genome level of DENV was determined by real time PCR. Cells were infected or mock infected with or without treatment with GA or vehicle as appropriate according to the standard protocol and levels of DENV genome were assessed at 48 h.p.i. Results (Fig. 11F) showed a slight but not significant reduction in cells treated with either GA or vehicle as compared to infection without treatment.

## Discussion

This study aimed to systematically survey the interaction of all ten DENV proteins with Hsp90. We were unable to source a commercially available antibody for NS2A protein. As for prM and NS4A proteins, no signal was detected when using the specific antibodies. For the remaining 7 proteins, interactions with Hsp90 were confirmed for 6, with only capsid protein showing no interaction with Hsp90. The remaining proteins, E, NS1, NS2B, NS3, NS4B and NS5 all interacted with Hsp90, although for NS1 we were only able to confirm the interaction in HepG2 cells. This may relate to the levels of NS1 in the two cell lines. HepG2 cells were derived from a liver hepatocellular carcinoma, while HEK293T/17 cells were derived from 293T cells, a derivative of 293 cells which were originally derived from human embryonic kidney cells. The liver has been well established as a target organ for DENV infection^30^. However, while HEK293T/17 is not an ideal model cell line for DENV infection, it can be both infected with DENV and can be used to obtain high levels of transfection as shown in our previous studies^9,31^, and as such its advantages broadly outweigh the fact that kidney cells are not a significant target of DENV infection^32^. In this case, as it is well characterized that NS3 and NS5 interact^29^, we transfected HEK293T/17 cells separately with constructs for each of these proteins (NS3 and NS5) to show that both, independently interacted with Hsp90.

Colocalization data obtained through co-immunoflourescence staining showed that there was colocalization between Hsp90 and several DENV proteins as would be expected given the broad interaction between Hsp90 and DENV proteins as shown here. However, there were differences in the degree of colocalization observed. In particular highest levels were observed between Hsp90 and NS1 and NS3, while lower levels of colocalization were observed between Hsp90 and DENV E protein, and between Hsp90 and NS5.

Hsp90 is predominantly characterized as a cytoplasmic protein, with its localization being mediated by sequences in the C-terminal half of the protein^33^, which was consistent with the differential colocalization with DENV NS5 observed with different anti-DENV NS5 antibodies. While DENV NS5 localizes in the cytoplasm as part of the replication complex, NS5 has a nuclear localization signal that mediates its translocaltion to the nucleus. Our observations showed no colocalization between nuclear NS5 and Hsp90, but colocalization between these two proteins occurred in the cytoplasm. In this regards, the sole DENV non-Hsp90 interacting protein we were able to identify (capsid) also contains a nuclear localization signal that mediates its translocation to the nucleus, resulting in distribution of capsid protein in both the nucleus and the cytoplasm^34^.

Perhaps the most surprising observation was that despite the interaction of Hsp90 with multiple DENV proteins, inhibition of Hsp90 activity using geldanamycin resulted in surprisingly small effects upon the DENV replication cycle. Indeed, inhibition of Hsp90 activity resulted in an increase in export of virus particles from the cell, as evidenced by increased virus output, and decreased levels of DENV E protein in the cell. While studies have shown that inhibition of Hsp90 can result in ubiquitin-mediated proteasomal degradation of client proteins^35^, little evidence of an effect on DENV proteins was observed. Indeed the reduction in cellular E protein levels was accompanied by an increase in both virus titer and the presence of E protein in the medium, suggesting that increased degradation of E protein was not a predominant process upon inhibition of Hsp90. Collectively these results suggest that Hsp90 inhibits to a small degree the formation or egress of the DENV viral particle. In contrast to studies with other viruses which have shown that Hsp90 can act as a pro-viral factor^24–28^, our results are consistent with Hsp90 having a slight anti-viral effect in DENV infection.

At this point we are unable to rule out other, non-chaperone related functions of Hsp90 in DENV replication as repeated attempts to down-regulate Hsp90 through siRNA mediated inhibition of gene expression were unsuccessful. This is possibly a consequence of the extremely high abundance of Hsp90 within the cell. However, the large number of interactions between Hsp90 and DENV proteins suggests Hsp90 may play other as yet undetermined roles in DENV replication.

## Materials and Methods

### Cells and virus

HEK293T/17 (human embryonic kidney) cells were cultured in Dulbecco’s minimal essential medium (DMEM, Gibco, Invitrogen, Carlsbad, CA) supplemented with 10% heat-inactivated fetal bovine serum (FBS, Gibco, Invitrogen) and incubated at 37 °C with 5% CO_2_. The cell line HepG2 (human hepatocellular carcinoma) was cultured in DMEM supplemented with 10% heat inactivated fetal bovine serum (FBS), 100 U/ml penicillin and 100 µg/ml streptomycin and incubated at 37 °C with 5% CO_2_. Dengue virus serotype 2 (DENV 2; strain 16681) was propagated in C6/36 cells as described elsewhere^36^. The supernatant containing progeny virus was harvested and supplemented with 20% heat-inactivated FBS and stored at −80 °C. DENV2 was titered by standard plaque assay on LLC-MK_2_ (Rhesus monkey kidney) cells essentially as described elsewhere^36^.

### Virus infection

On the day prior infection, HEK293T/17 or HepG2 cells as appropriate were seeded into culture plates under standard growth conditions which allowed 70–80% confluence to be reached within 24 hrs. After 24 hrs of cultivation, the culture medium was removed and the cells were incubated with DENV 2 in DMEM medium at a multiplicity of infection (m.o.i.) of 1 for HEK293T/17 or 20 for HepG2, or with DMEM alone (mock-infection) for 2 hours. Then the medium containing the virus was removed and replaced with fresh culture medium and the cells were further incubated under standard conditions for the indicated times.

### Western blot analysis

Mock infected and DENV 2 infected HEK293T/17 cells at the appropriate time point of post-infection were lysed using lysis buffer (50 mM Tris-HCl (pH 7.5), 150 mM NaCl, 1% NP-40, 0.5 mM EDTA, 0.5 mM activated Na_3_VO_4_ and 1X protease inhibitor cocktail (PIC)) followed by vortexing. The lysates were subsequently centrifuged at 16,000 × g for 5 min to remove cell debris, and after transferring the supernatant to a new tube the protein concentration was determined using the Bradford assay. A total of 30 µg proteins were separated through 10% sodium dodecyl sulfate polyacrylamide (SDS-PAGE) gels, and proteins were subsequently electrophoretically transferred to nitrocellulose membranes. The membranes were blocked with 5% skim milk in TBS/0.1% Tween-20 for 2 hours at room temperature, following which they were incubated overnight with an appropriate primary antibody at 4 °C followed by incubation with an appropriate secondary antibody conjugated with HRP. Primary and secondary antibodies used in western analysis with details on vendor and dilution are listed in Supplementary Table S1. Signals were developed using Amersham ECL Plus Western Blotting Detection reagents (GE Healthcare) used in accordance to the manufacturer’s instructions. The signal was then captured by autoradiography film or by an Azure c400 visible fluorescent western blot imaging system (Azure Biosystems).

### Co-immunoprecipitation (Co-IP) and reverse Co-IP assays

HEK293T/17 cells or HepG2 cells as appropriate were grown in 60 mm tissue culture dishes. Mock infection, DENV infection, mock transfection, and transfection were carried out according to the standard protocol. At 2 days post-infection or transfection, the cells were harvested and proteins were extracted using the same protocol as for western blot analysis. A total of 1 mg of cell lysate was pre-cleared by incubation with Protein G Sepharose 4 Fast Flow media (GE Healthcare, Buckinghamshire, UK) at 4 °C and with rotatation for 1 hr. Subsequently, 100 µl of pre-cleared lysates from infections or mock-infections were incubated with or without the pull down antibody given in Supplemental Table S2, with gentle end-over-end mixing at 4 °C overnight. Subsequently, the mixture was incubated with 30 µl of a 50% slurry of protein G sepharose beads with gentle end-over-end mixing at 4 °C for 6 hr. Then the mixture was centrifuged at 6,000 × *g* for 3 min at 4 °C and the supernatant was discarded. The pellet was washed three times with lysis buffer without EDTA or NP40, and finally resuspended in 30 µl of 3 × SDS sample buffer. The samples were heated at 100 °C for 5 min followed by centrifugation at 14,000 × g for 3 min at 4 °C. The supernatant was collected and proteins were separated by electrophoresis through 10% SDS-PAGE gels. After electrophoresis, proteins were transferred to solid matrix for western blotting. After blocking membranes were incubated overnight at 4 °C with the primary antibodies shown in Supplemental Table S2, followed by an appropriate secondary antibody (Supplemental Table S2) for 2 hr. Finally, the signal was developed as described for western blotting. For reverse co-immunoprecipitation, one of the antibodies listed in Supplemental Table S3 was used as the pull down antibody, with the resultant filters being probed with the primary and secondary antibodies listed in Supplementary Table S3.

### Calcium phosphate transfection

Prior to transfection, HEK293T/17 cells were plated into 100 mm tissue culture dishes at a density that allowed 80% confluence to be reached on the day of transfection. The cells were transfected with eukaryotic expression plasmids containing DENV NS3 or NS5 (pCDNA_ D2NS3, pEGFPC2_D2NS5) or with pCDNA_EGFP or pEGFPC2 essentially as described elsewhere^9^. Briefly 15 µg of each plasmid was diluted in 500 µl of Opti-MEM (Invitrogen), 500 µl of 2X HBS and 50 µl of 2.5 M CaCl_2_ and the mixture was incubated for 20 min at room temperature. Then the plasmid solution was added drop-wise into each dish of cells with gentle mixing. Transfected cells were incubated at 37 °C with 5% CO_2_. Media was changed at 20 hours post transfection (h.p.t.) and cells were harvested at 48 h.p.t.

### Immunofluorescence assay

HEK293T/17 cells were grown on cover slips in 24 well tissue culture plates until 60% confluence. Then the cells were infected with DENV 2 at moi of 1. At 2 d.p.i, the cell culture media was removed and cells were washed with 1XPBS. The cells were fixed with 100% ice-cold methanol for 10–15 min at room temperature and allowed to dry for 15 min. Next, the cells were washed twice with 1XPBS/IFA for 5 min and permeabilized with 0.3% Triton-X 100 in 1X PBS/IFA for 10 min followed by washing twice with 0.03% Triton-X 100 for 5 min. The cells were blocked with 5% BSA in 1X PBS/IFA for 1 hr. After that, the cells were incubated with an appropriate primary antibody (Supplemental Table S4) at 4 °C for overnight. After washing, the cells were incubated with an appropriate secondary antibody (Supplemental Table S4), and a 1:200 dilution of 4′ 6-diamidino-2-phenyllindole (DAPI) at room temperature for 1 hr in the dark. After that the cells were washed six times with 0.03% Triton-X 100 for 5 min followed by mounting the cover slips onto glass slides using Prolong® Gold antifade reagent. The signals were observed under Olympus fluorescent confocal microscope.

### Cell viability assay for geldanamycin treatment

HEK293T/17 cells were seeded into the wells of a ninety-six well tissue culture plate and incubated under standard conditions until the cells were 90% confluent. Then, the cell culture media was removed and cells were incubated with geldanamycin (GA) at concentrations of 20, 50, 100, 150, 200, 300, 500 and 800 nM for 24 and 48 hr. Next, the cell culture media was removed and the cells were incubated with 1X Presto Blue reagent diluted with complete medium at 37 °C, 5% CO_2_ for 1 hr. Finally, fluorescence was measured at excitation 535 nm and emission 595 nm. In addition, cellular morphology was observed under a light microscope.

### Hsp90 inhibitor preparation and treatment

Geldanamycin (GA; Merck & Co., Inc., Kenilworth, NJ) was dissolved in 100% DMSO. Solutions containing 300 nM of GA were freshly prepared with complete DMEM containing 10% FBS. HEK293T/17 cells were grown in 100 mm^2^ tissue culture dishes under standard conditions until 90% confluence, after which the cells were treated with GA at a concentration of 300 nM for 2 hr. The media was subsequently removed and cells were mock infected or infected with DENV 2 at moi 1 for 2 hr in the absence of the drug. After 2 hr, the media was removed and replaced with fresh medium containing GA. Cells were incubated at 37 °C, 5% CO_2_ for 48 hrs.

### Flow cytometry

DENV infected or mock infected cells were washed with 1X PBS followed by fixation with 4% paraformaldehyde in 1X PBS for 20 min at room temperature in dark. Then the cells were washed twice with 1% BSA in 1X PBS and centrifuged at 6,000 × g for 5 min. the cells were permeabilized with 0.2% Triton x-100 for 10 min in the dark at room temperature. Subsequently, the cells were washed with 1% BSA in 1X PBS followed by incubation with a 1:150 dilution of monoclonal antibody HB114^37^ diluted in 1% BSA in 1X PBS at 4 °C overnight with constant agitation. The cells were washed with 1% BSA in 1X PBS and incubated with 1:40 dilution of a goat polyclonal anti-mouse IgG antibody conjugated with FITC (02-18-06; KPL, Guilford, UK) for 1 hr at room temperature in dark, after which the cells were washed with 1% BSA in 1X PBS. The fluorescent signal was determined by flow cytometery (BD FACSCalibur) using the CELLQuest^TM^ software. All experiments were performed as three independent replications.

### RNA extraction and real time PCR

RNA was extracted from cells using Trizol reagent (Molecular Research Center, Cincinati, OH). Random hexamers were used to synthesize the first strand cDNA using ImpromII^TM^ reverse transcriptase. After that the samples were subjected to real time PCR using specific primers for the DENV genome and actin as an internal control. The PCR reaction contained 1 μg of DNA template, 0.1 μM of each primer (NS1fwd: 5′-TGCTGACATGGGTTATTGGATAG-3′ and NS1rev: ACTCCATTGCTCCACAGTGTGTG-3′; β-actinF 5′GAAGATGACCCAGATCATGT3′ and β-actinR 5′ATCTCTTGCTCGAAGTCCAG3′), SYBR green fluorescent dye (KAPA SYBR® FAST ABI Prism® qPCR kit, Kapa Biosystems Inc, MA, USA) The cycle conditions were 95 °C for 3 min, followed by 40 cycles of 95 °C for 10 sec, 60 °C for 30 sec and extension of 72 °C for 20 sec.

### Data availability statement

All data generated or analysed during this study are included in this published article (and its Supplementary Information files).

## Electronic supplementary material

Supplementary Files
