# Supplementary material for: Hsp90 interacts with multiple dengue virus 2 proteins
Source: Sci Rep. 2018 Mar 9;8:4308. doi: 10.1038/s41598-018-22639-5 (PMC5844963; doi:10.1038/s41598-018-22639-5)
Supplement: Supplementary file 1 — Supplementary Files [file 41598_2018_22639_MOESM1_ESM.pdf]

Supplementary materials

Hsp90 interacts with multiple dengue virus 2 proteins

Kanjana Srisutthisamphan[1], Krit Jirakanwisal[1], Suwipa Ramphan[1], Natthida Tongluan[1], Atichat Kuadkitkan[1], Duncan R. Smith[1]*

[1]Institute of Molecular Biosciences, Mahidol University, Bangkok, Thailand

*Correspondence to:

Duncan R. Smith, Institute of Molecular Biosciences, Mahidol University, Salaya Campus, 25/25 Phuttamontol Sai 4, Salaya, Nakorn Pathom, Thailand 73170.

Tel (662) 800 3624-8; Fax (662) 4419906; Email: duncan_r_smith@hotmail.com

Table S1. Antibodies used in western blots

Name of antibody	Source	Type	Dilution	Detail
Pan specific anti-dengue virus type 1-4 antibody (MA1-27093; Pierce, Rockford, IL, USA)	mouse	P	1:500	1°Ab
Anti-dengue NS3 protein antibody (PA5-32199; Pierce, Rockford, IL, USA)	rabbit	P	1:3000	1°Ab
Anti-dengue NS5 protein antibody (GTX629446; GeneTex Inc, CA, USA)	mouse	M	1:3000	1°Ab
Anti-Hsp90α/β (H-114): (sc-7947) Santa Cruz, Biotechnology Inc., Texas, USA	rabbit	P	1:10000	1°Ab
Anti actin protein (sc-1616; Santa Cruz, Biotechnology Inc., Texas, USA)	goat	P	1:10000	1°Ab
HRP-conjugated rabbit anti-mouse IgG (A9044; Sigma, MO, USA)	rabbit	P	1:8000	2°Ab
HRP-conjugated goat anti-rabbit IgG (31460; Pierce, IL, USA)	goat	P	1:10000	2°Ab
HRP-conjugated rabbit anti-goat IgG (31402; Pierce, IL, USA)	rabbit	P	1:10000	2°Ab

Table S2. Antibodies used in co-immunoprecipitation assays

Name of antibody	Source	Dilution /concentration	Use
Anti-Hsp90α/β (H-114): (sc-7947, Santa Cruz, Biotechnology Inc., Texas, USA)	rabbit	1 μg	Pull down
Pan specific anti-dengue virus type 1-4 antibody (MA1-27093; Pierce, Rockford, IL, USA)	mouse	1:500	1°Ab
Anti-Dengue virus prM glycoprotein antibody (ab41473, Abcam plc, Cambridge, UK)	mouse	1:500	1°Ab
Anti-Dengue virus NS1 glycoprotein antibody (ab41616, Abcam plc, Cambridge, UK)	mouse	1:500	1°Ab
Anti-NS2B antibody (GTX124246; GeneTex Inc, CA, USA)	rabbit	1:3000	1°Ab
Anti-dengue NS3 protein antibody (PA5-32199; Pierce, Rockford, IL, USA)	rabbit	1:3000	1°Ab
Dengue virus type 2 NS4A Antibody (PA5-32197; Pierce, Rockford, IL, USA)	rabbit	1:500	1°Ab
Anti-dengue NS4B protein antibody (PA5-32198; Pierce, Rockford, IL, USA)	rabbit	1:3000	1°Ab
Anti-dengue NS5 protein antibody (GTX629446; GeneTex Inc, CA, USA)	mouse	1:3000	1°Ab
Anti-Dengue virus 2 antibody (capsid) (ab155042, Abcam plc, Cambridge, UK)	rabbit	1:2000	1°Ab
Anti-Hsp70 (K-20-R, Santa Cruz, Biotechnology Inc., Texas, USA)	rabbit	1:3000	1°Ab
HRP-conjugated goat anti-rabbit IgG (31460; Pierce, IL, USA)	goat	1:8000	2°Ab
HRP-conjugated rabbit anti-mouse IgG (A9044; Sigma, MO, USA)	rabbit	1:8000	2°Ab

Table S3. Antibodies used in reverse co-immunoprecipitation assays

Name of antibody	Source	Dilution /concentration	Use
Pan specific anti-dengue virus type 1-4 antibody (MA1-27093; Pierce, Rockford, IL, USA)	mouse	1 µg	Pull down
Anti-NS2B antibody (GTX124246; GeneTex Inc, CA, USA)	rabbit	1 µg	Pull down
Anti-dengue NS3 protein antibody (PA5-32199; Pierce, Rockford, IL, USA)	rabbit	1 µg	Pull down
Anti-dengue NS4B protein antibody (PA5-32198; Pierce, Rockford, IL, USA)	rabbit	1 µg	Pull down
Anti-dengue NS5 protein antibody (GTX629446; GeneTex Inc, CA, USA)	mouse	1 µg	Pull down
Anti-Dengue virus 2 antibody (capsid) (ab155042, Abcam plc, Cambridge, UK)	rabbit	1 µg	Pull down
Anti-Hsp90α/β (H-114): (sc-7947)	rabbit	1:10000	1°Ab
HRP-conjugated goat anti-rabbit IgG (31460; Pierce, IL, USA)	goat	1:10000	2°Ab
HRP-conjugated rabbit anti-mouse IgG (A9044; Sigma, MO, USA)	rabbit	1:8000	2°Ab

Table S4. Antibodies used in confocal microscopy

Name of antibody	Source	Type	Dilution	Detail
Pan specific anti-dengue virus type 1-4 antibody (MA1-27093; Pierce, Rockford, IL, USA)	mouse	P	1:100	1°Ab
Anti-dengue NS1 glycoprotein (PA5-32207; Pierce, Rockford, IL, USA)	rabbit	P	1:25	1°Ab
Anti-dengue NS3 protein antibody (PA5-32199; Pierce, Rockford, IL, USA)	rabbit	P	1:25	1°Ab
Anti-dengue NS5 (PA5-27888); Pierce, Rockford, IL, USA)	rabbit	P	1:50	1°Ab
Anti-dengue NS5 protein antibody (GTX629446; GeneTex Inc, CA, USA)	mouse	M	1:50	1°Ab
Anti-Hsp90α/β (H-114): (sc-7947) Santa Cruz, Biotechnology Inc., Texas, USA	rabbit	P	1:50	1°Ab
Anti-Hsp90α/β ((F-8): (sc-13119) Santa Cruz, Biotechnology Inc., Texas, USA	mouse	P	1:50	1°Ab
Alexa Fluor® 488 donkey anti-mouse IgG antibody (A11029, Invitrogen)	donkey	P	1:100	2°Ab
Rhodamine red™-X-conjugated goat anti-rabbit IgG antibody	goat	P	1:100	2°Ab

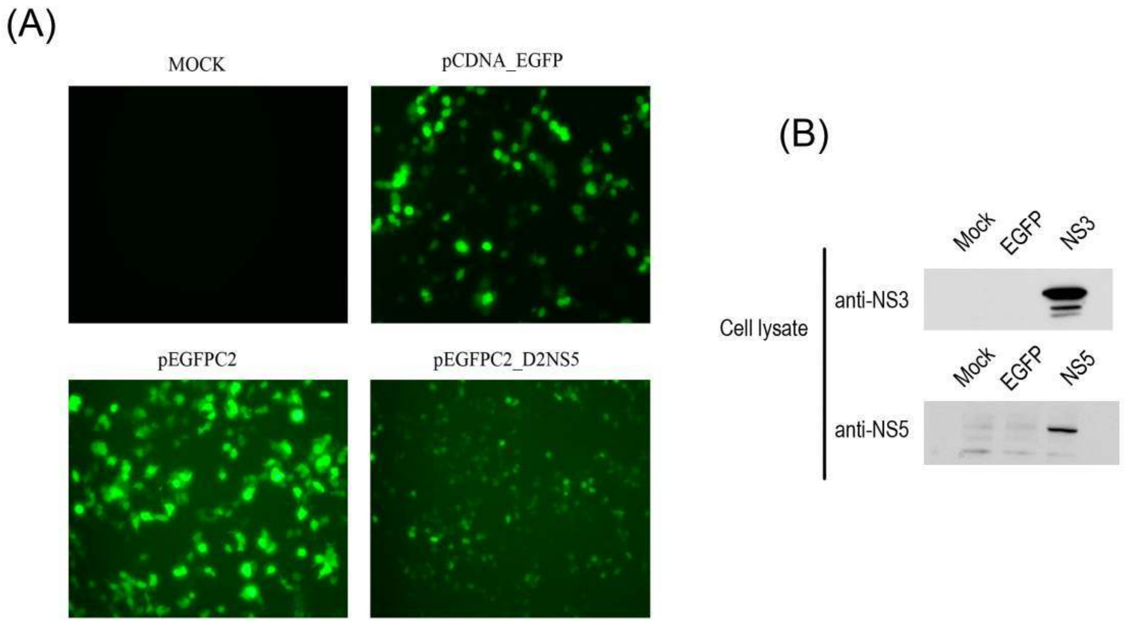

Supplemental Figure S1. The transfection efficiency of eukaryotic expression plasmid DNA and the expression of NS3 and NS5 protein from HEK293T/17 transfected cells.

(A) HEK293T/17 cells were transfected with eukaryotic expression plasmid DNA (pCDNA_D2NS3 or pEGFPC2_D2NS5) using the calcium phosphate transfection method. The green color represents GFP expression protein. The expression of GFP was examined for transfection efficiency at two days post transfection. (B) Proteins extracted from transfected HEK293T/17 cells and were separated by SDS-PAGE and then transferred to nitrocellulose membranes. The membranes were probed with a polyclonal NS3 antibody and monoclonal NS5 antibody, individually followed by appropriate secondary antibodies conjugated with HRP.

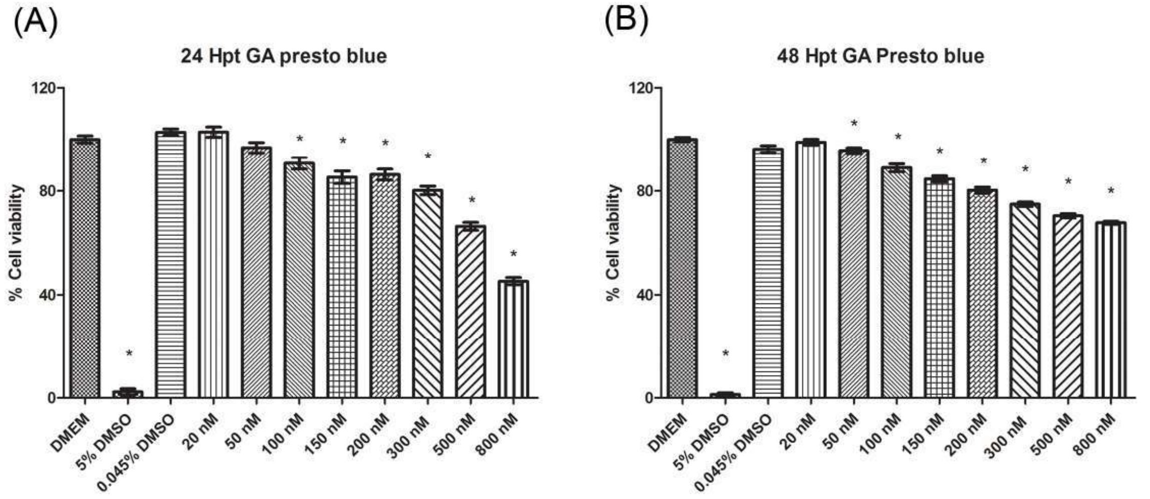

Supplemental Figure S2. Cytotoxicity analysis of geldanamycin.

HEK293T/17 cells were treated with geldanamycin at different concentration. The untreated cells (DMEM) and 5% DMSO treated cells were included as negative and positive controls, respectively. The results at 24 and 48 h.p.t (hours post treatment.) are shown in graph A and graph B, respectively. (*p-value < 0.05)

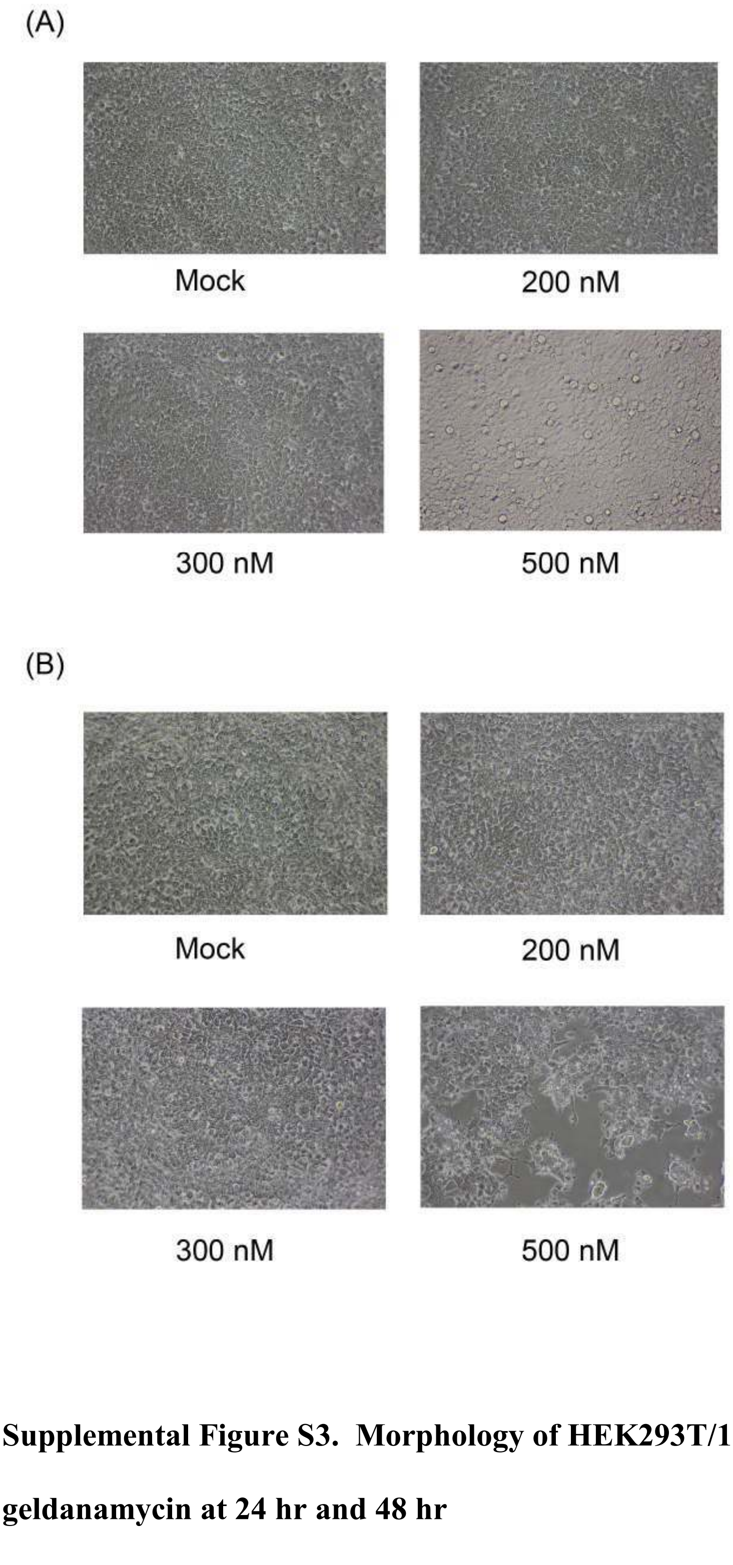

Supplemental Figure S3. Morphology of HEK293T/17 cells after treatment with geldanamycin at 24 hr and 48 hr

After treating with geldanamycin at concentrations 200, 300 and 500 nM or mock treating (vehicle only), the cells were observed under an inverted microscope at 24 (A) and 48 (B) hours post treatment (h.p.i.).

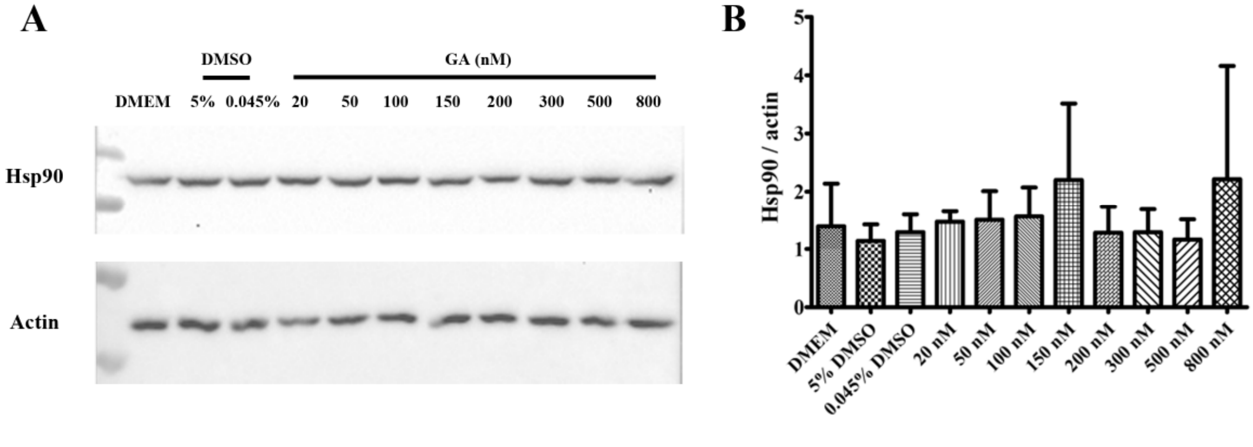

Supplemental Figure S4. Expression of hsp90 after treatment with geldanamycin

HEK293T/17 cells were treated with geldanamycin at concentrations up to 800 nM or mock treated, or treated with DMSO for 48 hours before (A) expression of Hsp90 was determined by western blot analysis. Filters were reprobed to determined actin expression. Experiment was undertaken independently in triplicate and representative blots are show. (B) Band intensities were determined by the Quantity One software and Hsp90/actin plotted for the three replicates. No statistical significance was observed between any groups. Error bars show S.E.M. Uncropped images can be found in the following supplemental materials.

Uncropped images

HEK293T cell

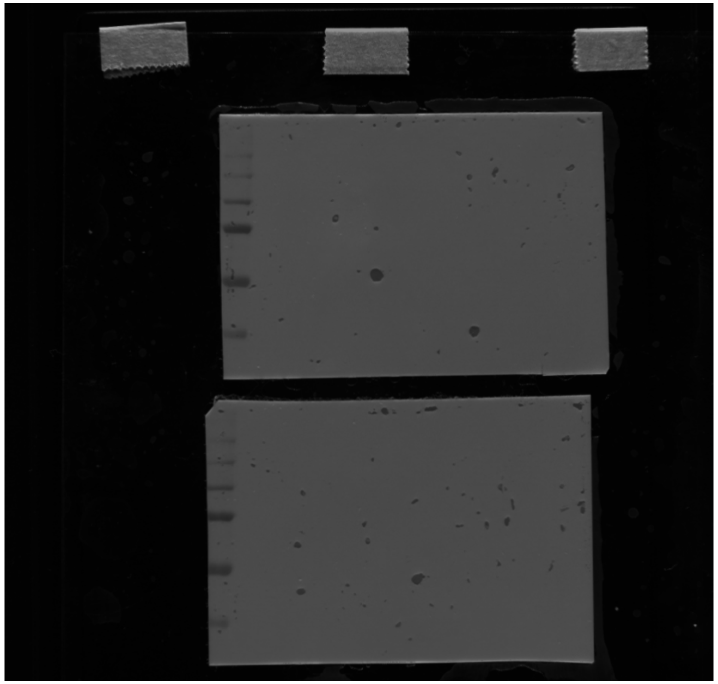

Figure 1 Hsp90 mock
upper membrane

Figure 1 Hsp90 infected
Lower membrane

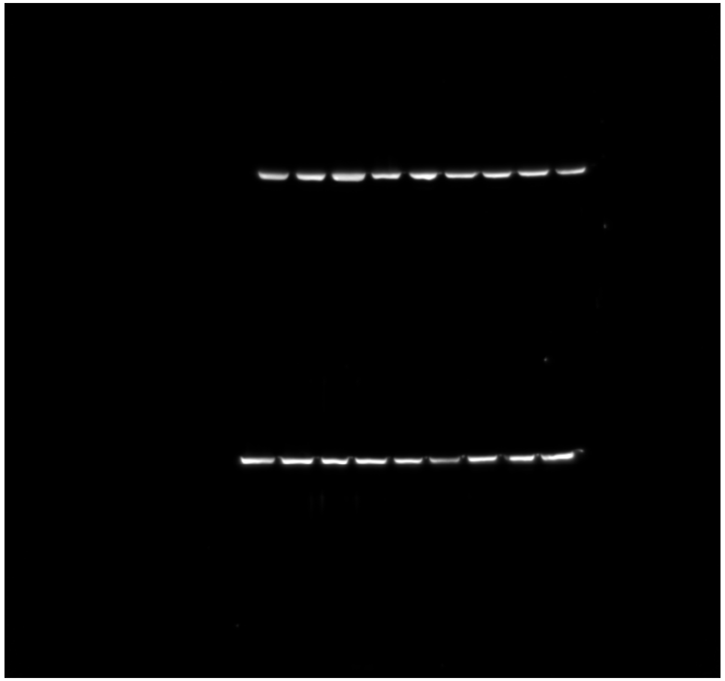

HEK293T cell

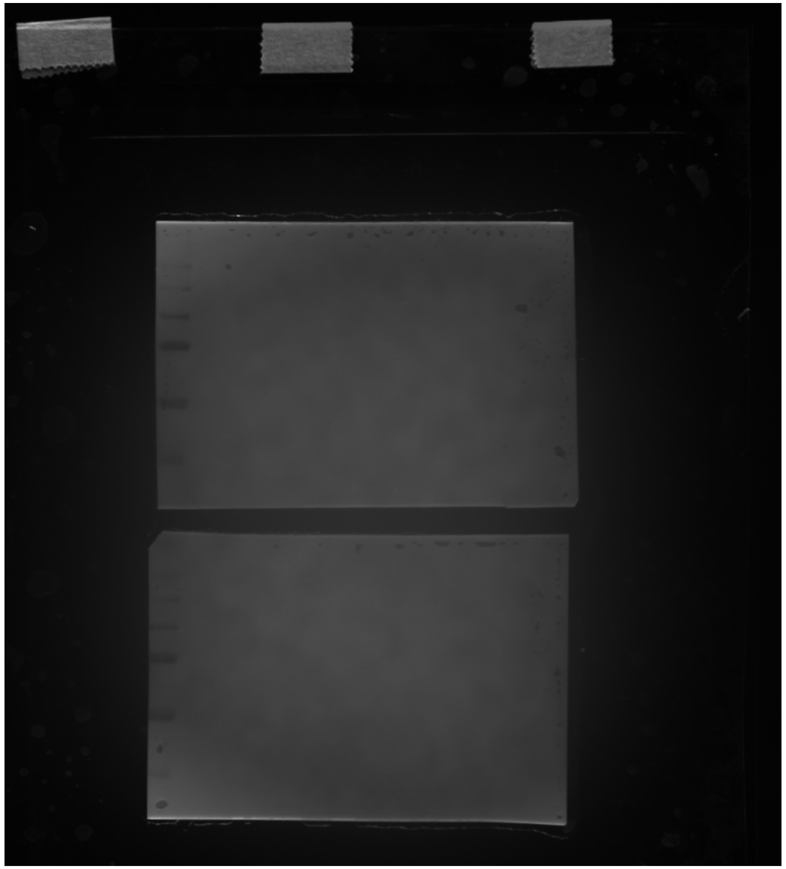

Figure 1 Actin mock
upper membrane

Figure 1 Actin infected
Lower membrane

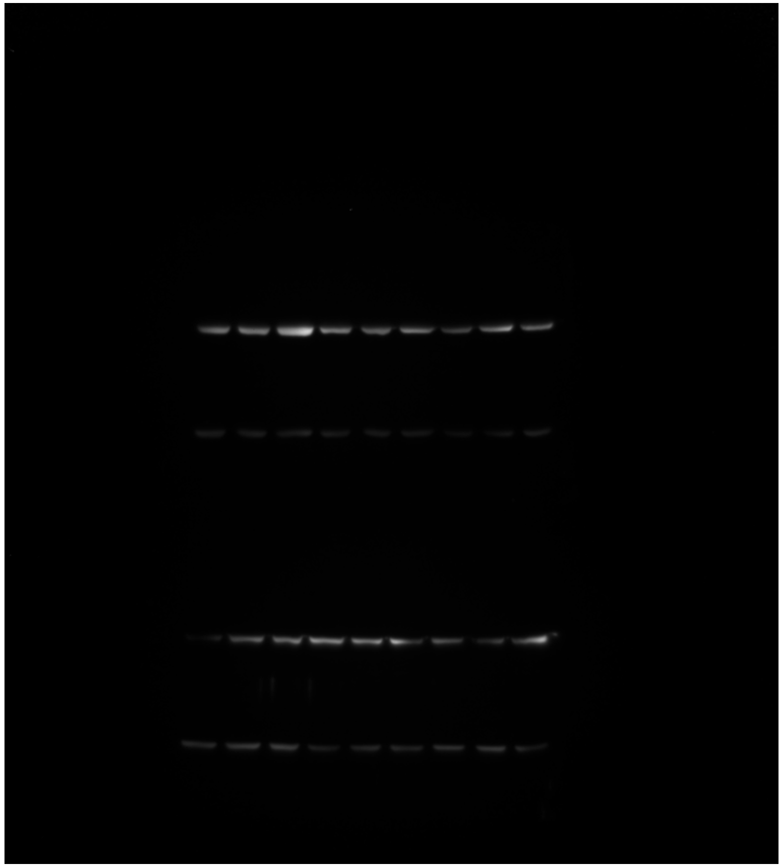

HEK293T cell

Figure 2: CO-IP probe E protein

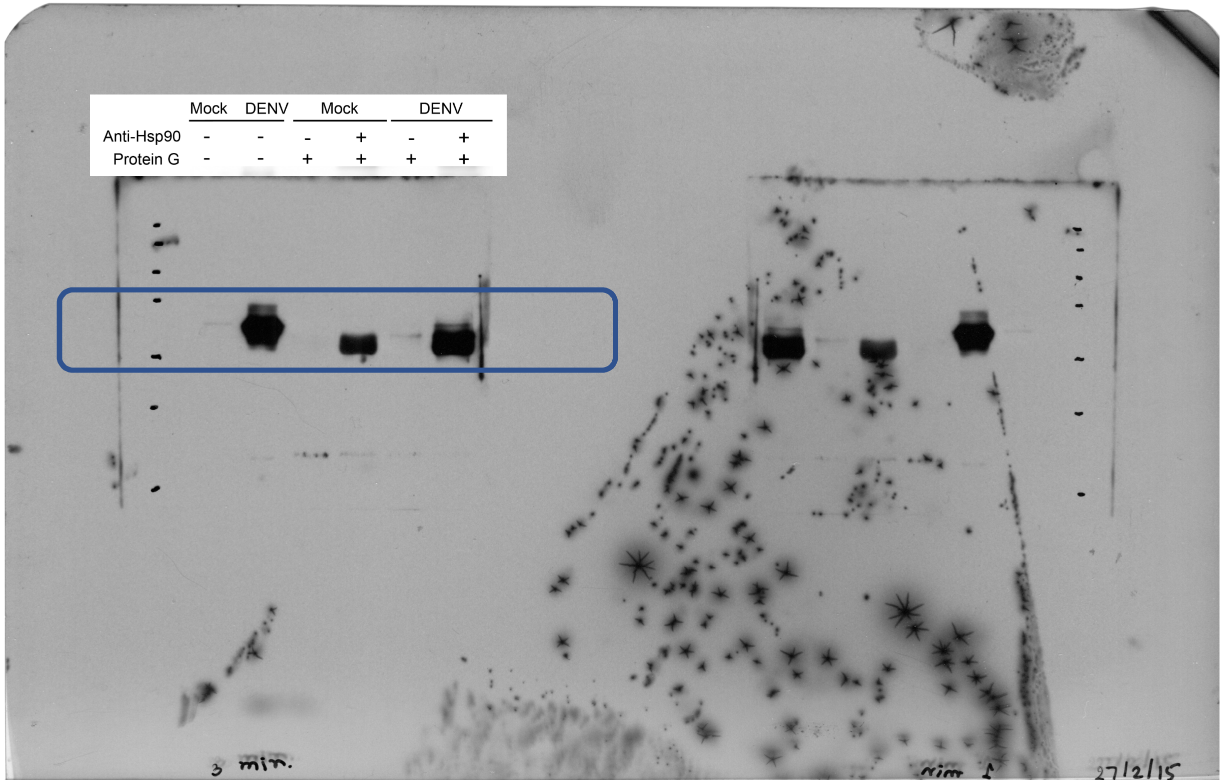

HEK293T cell

Figure 2: CO-IP probe NS2B protein

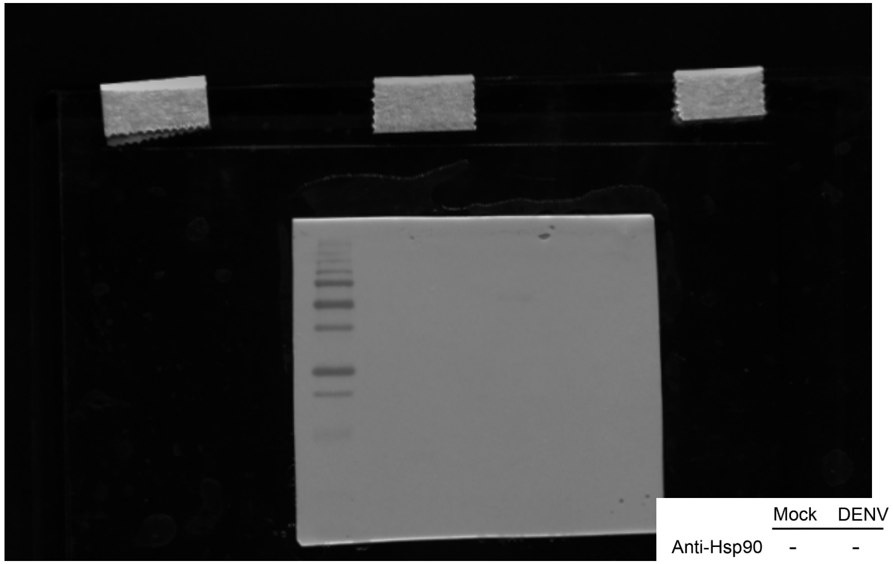

Bright field

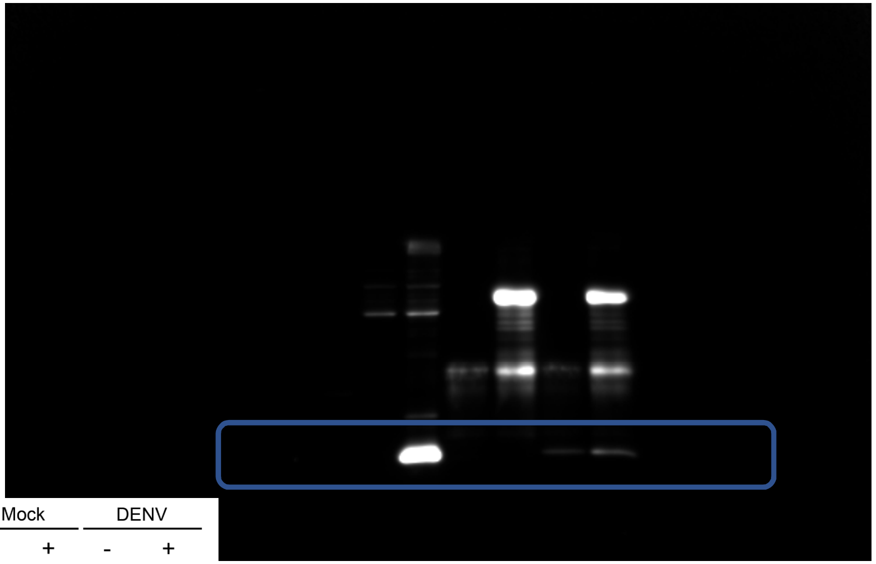

Expose field

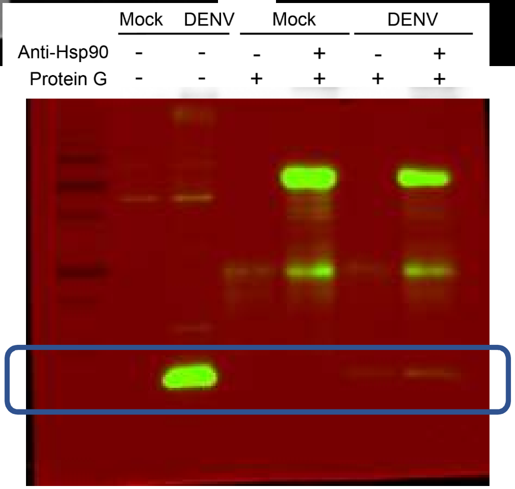

HEK293T cell

Figure 2: CO-IP probe NS3 protein

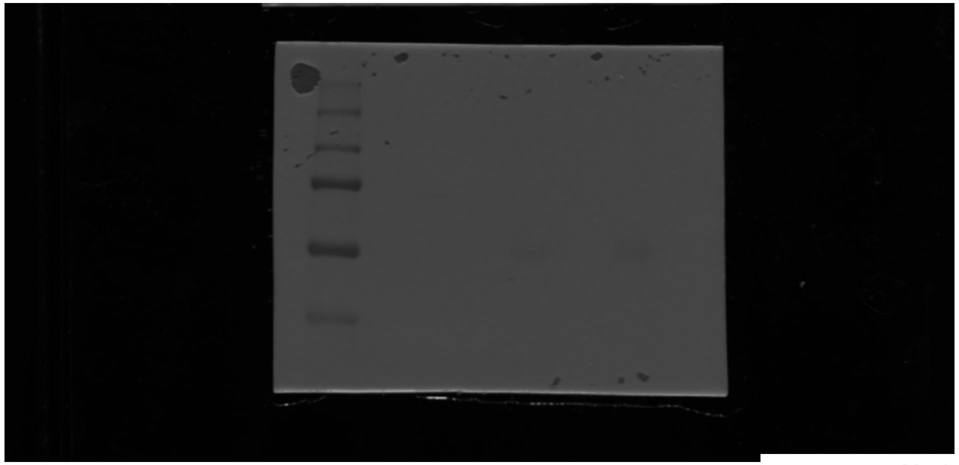

Bright field

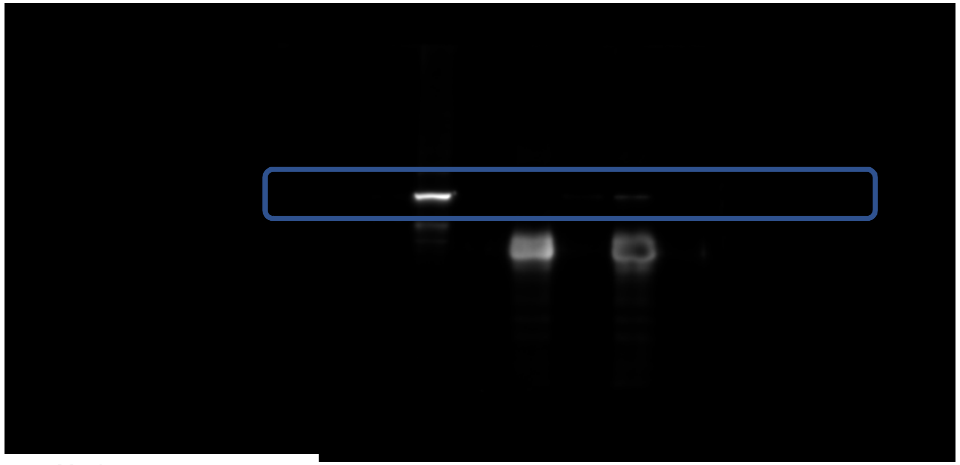

Expose field

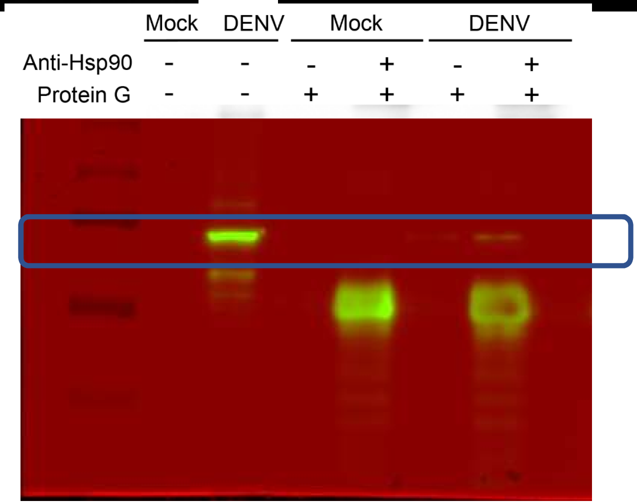

HEK293T cell

Figure 2: CO-IP probe NS4B protein

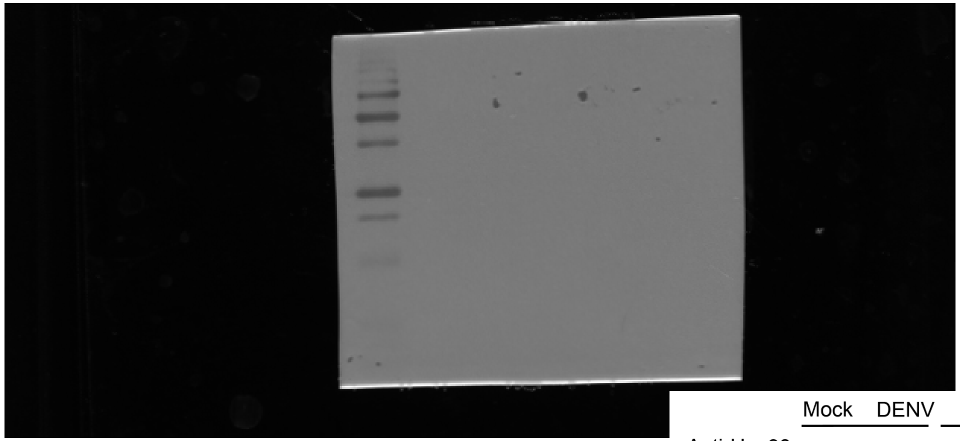

Bright field

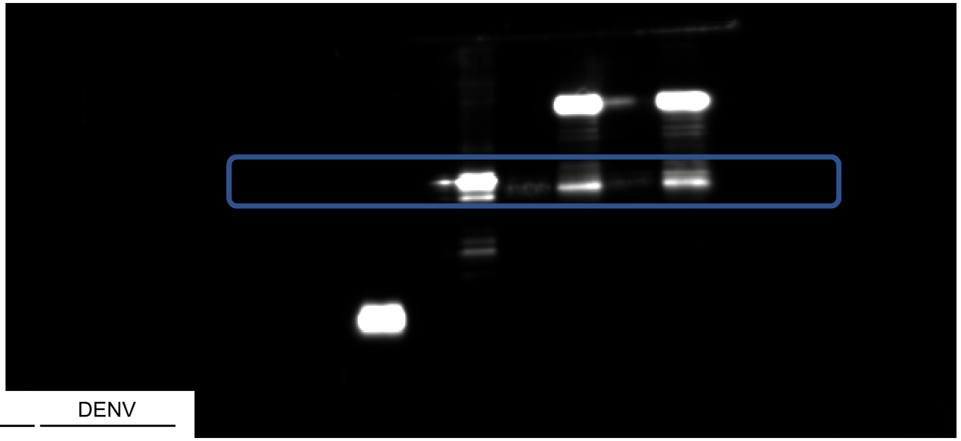

Expose field

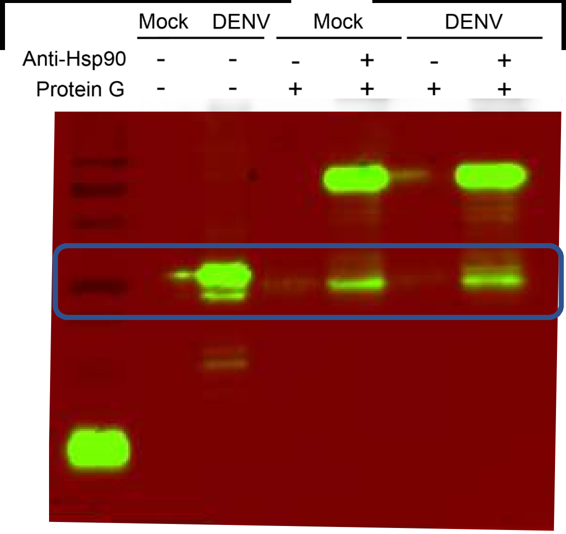

HEK293T cell

Figure 2: CO-IP probe NS5 protein

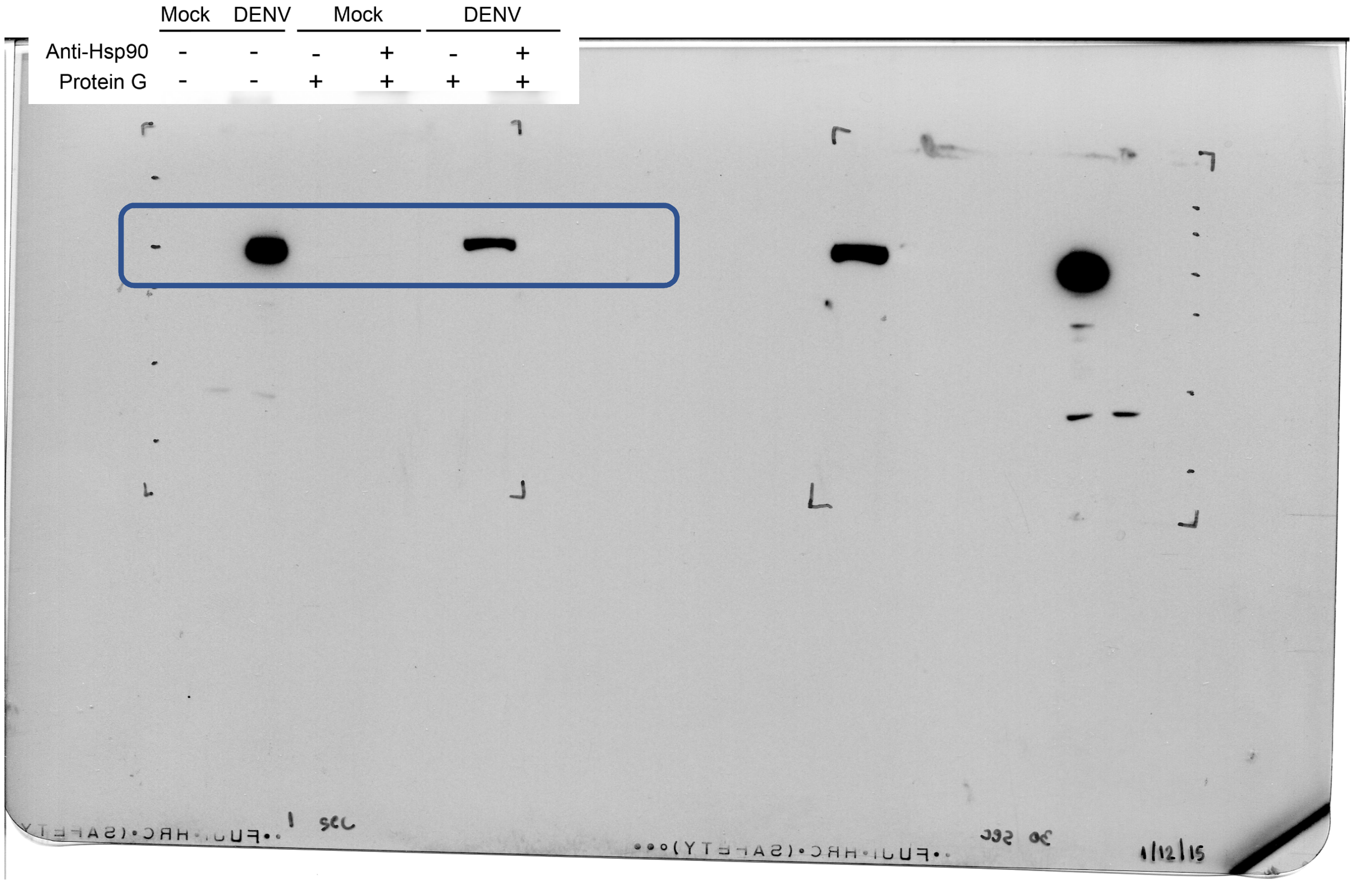

HEK293T cell

Figure 2: CO-IP probe capsid protein

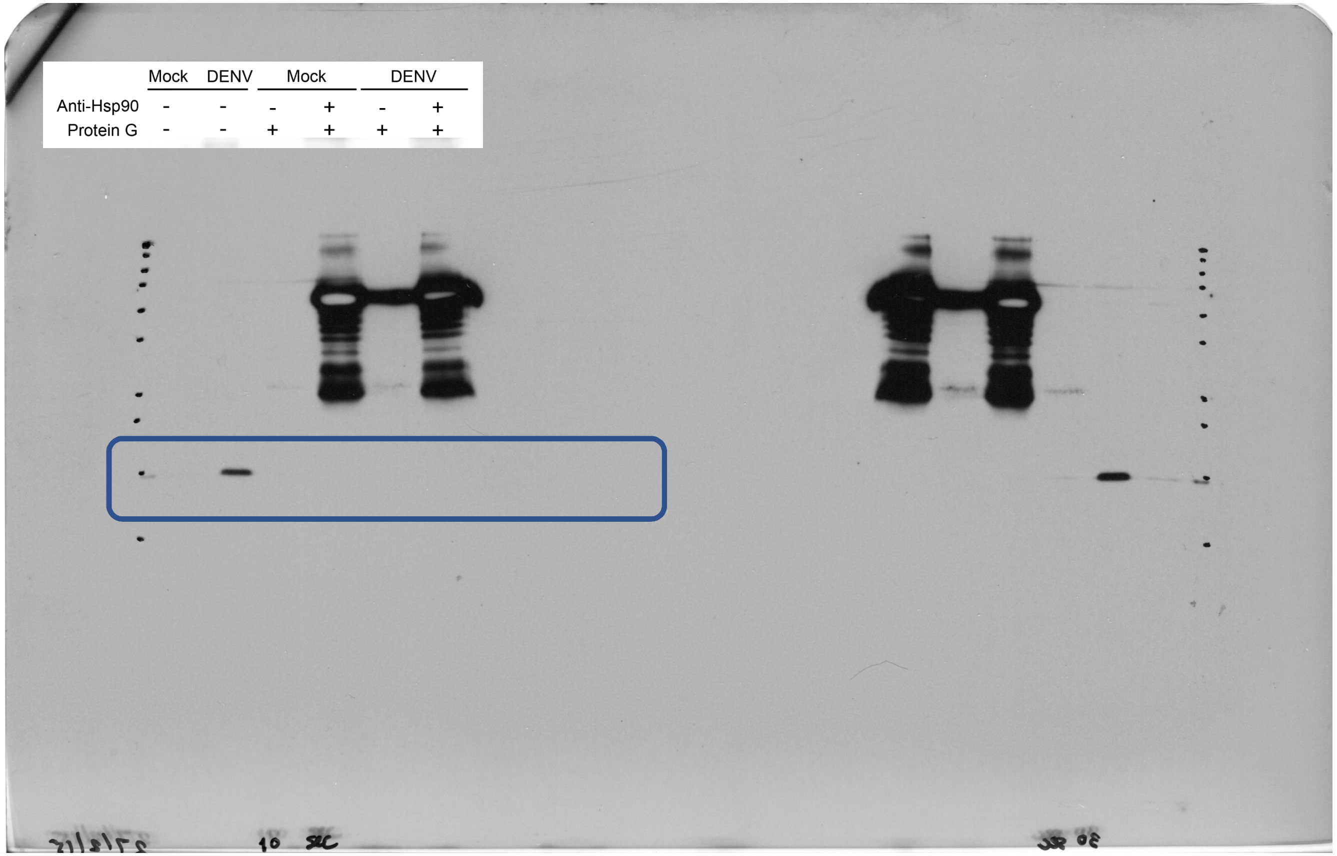

HepG2 cell

Figure 3: CO-IP probe E protein

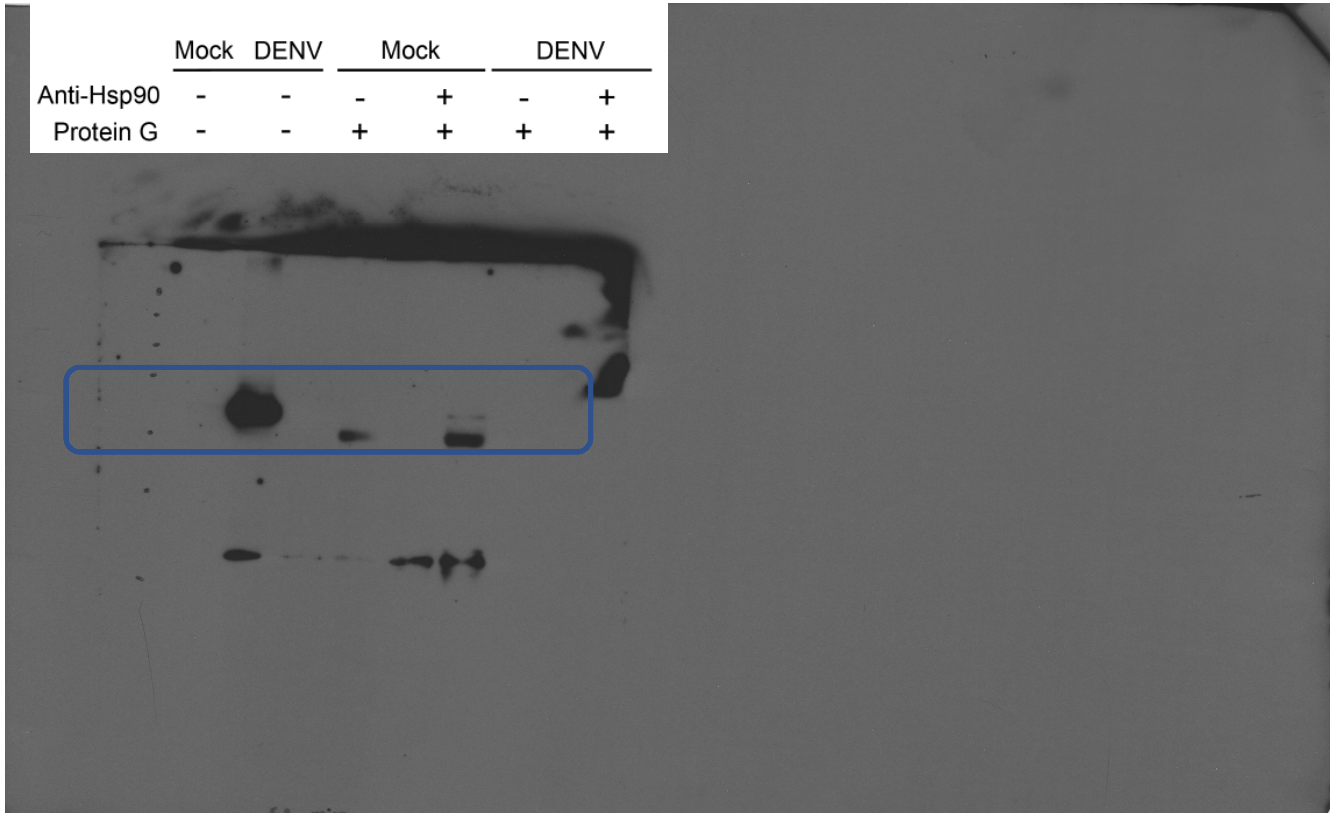

HepG2 cell

Figure 3: CO-IP probe NS1 protein

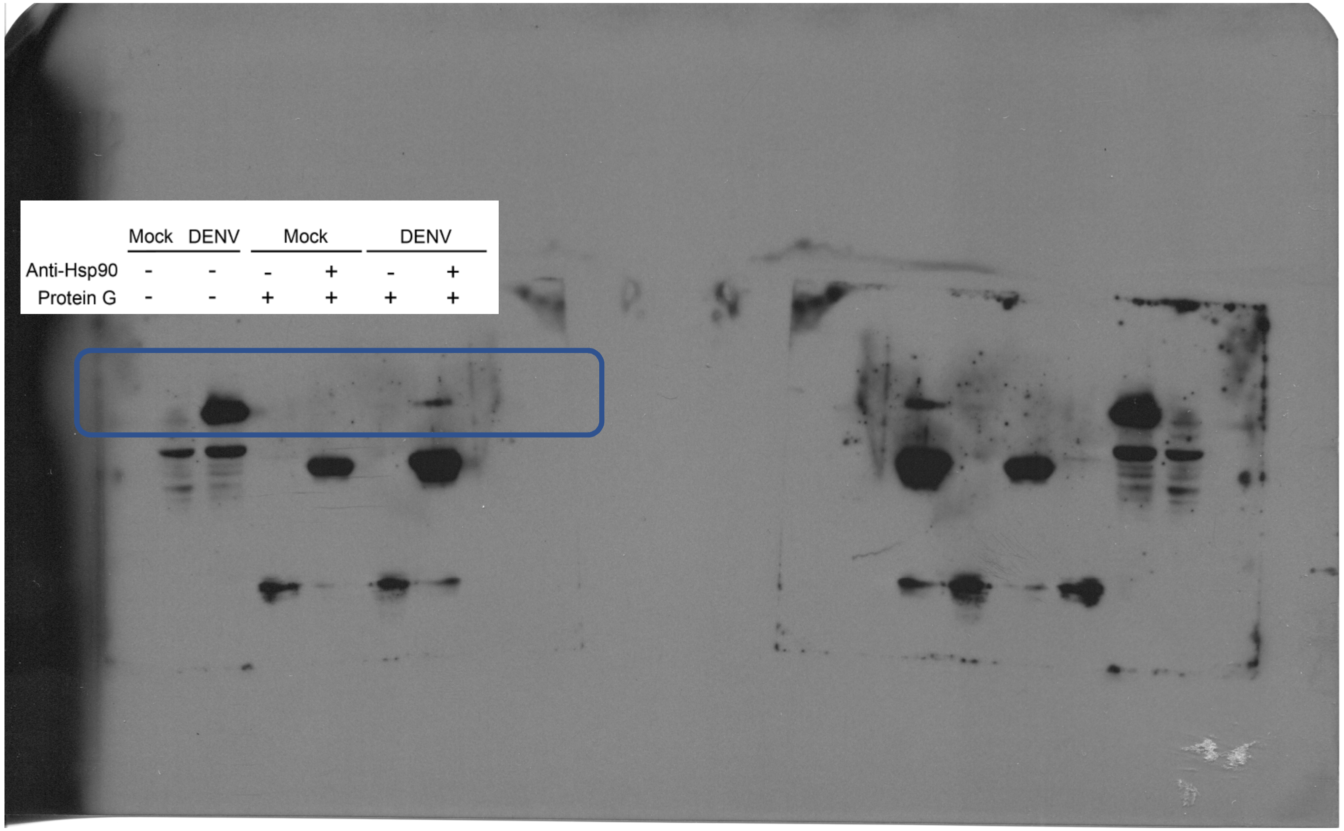

HepG2 cell

Figure 3: CO-IP probe capsid protein

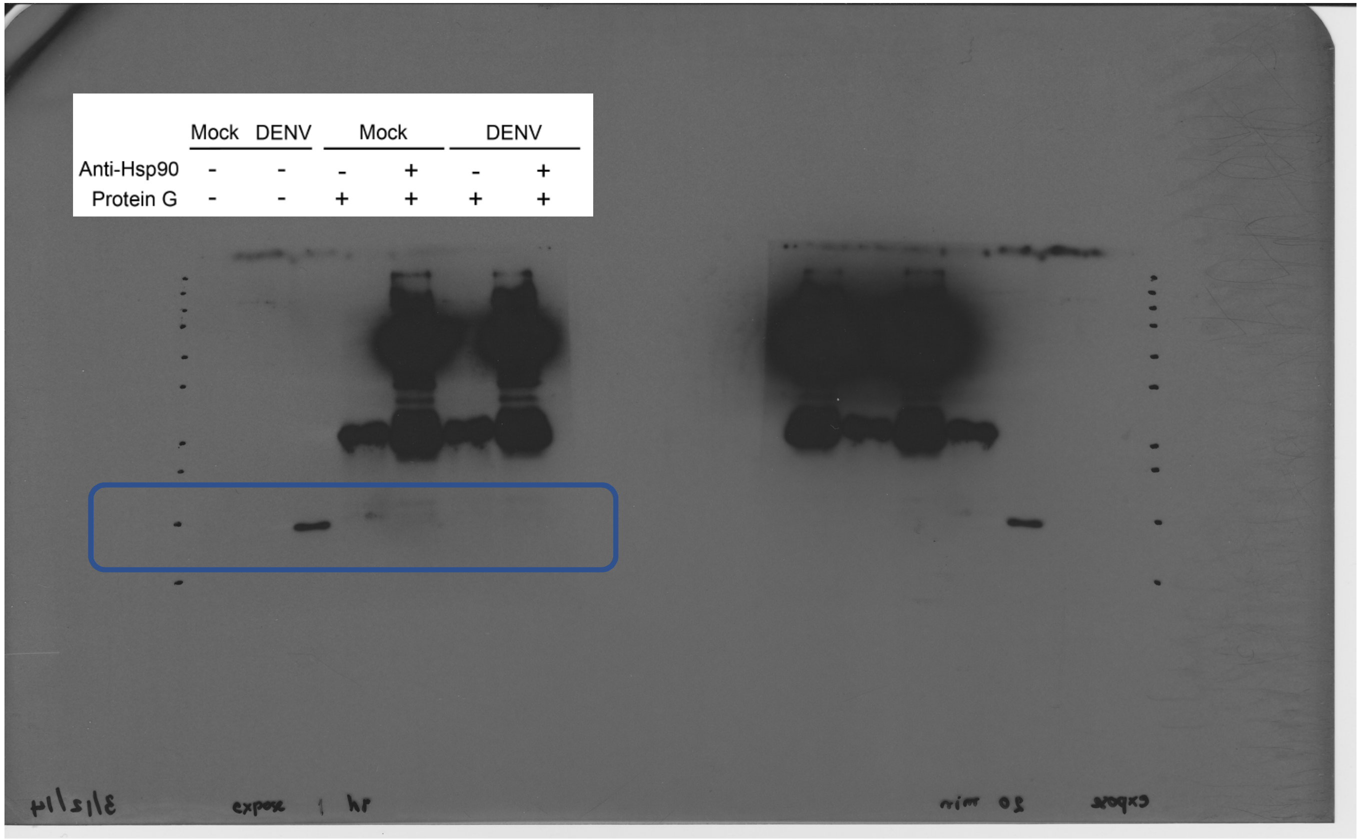

HepG2 cell

Figure 3: CO-IP probe Hsp70 protein

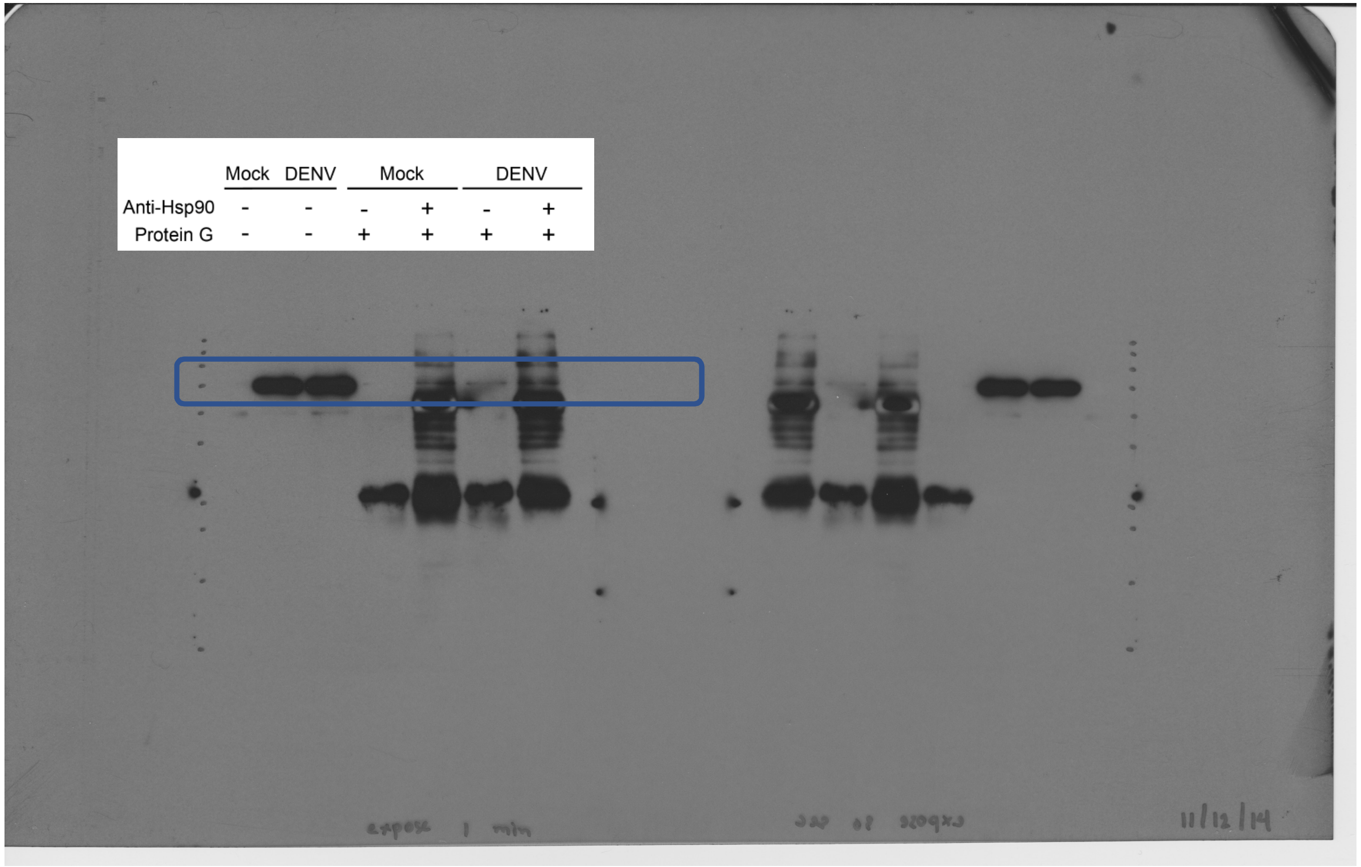

HEK293T cell

Figure 4: reverse CO-IP pull down E protein

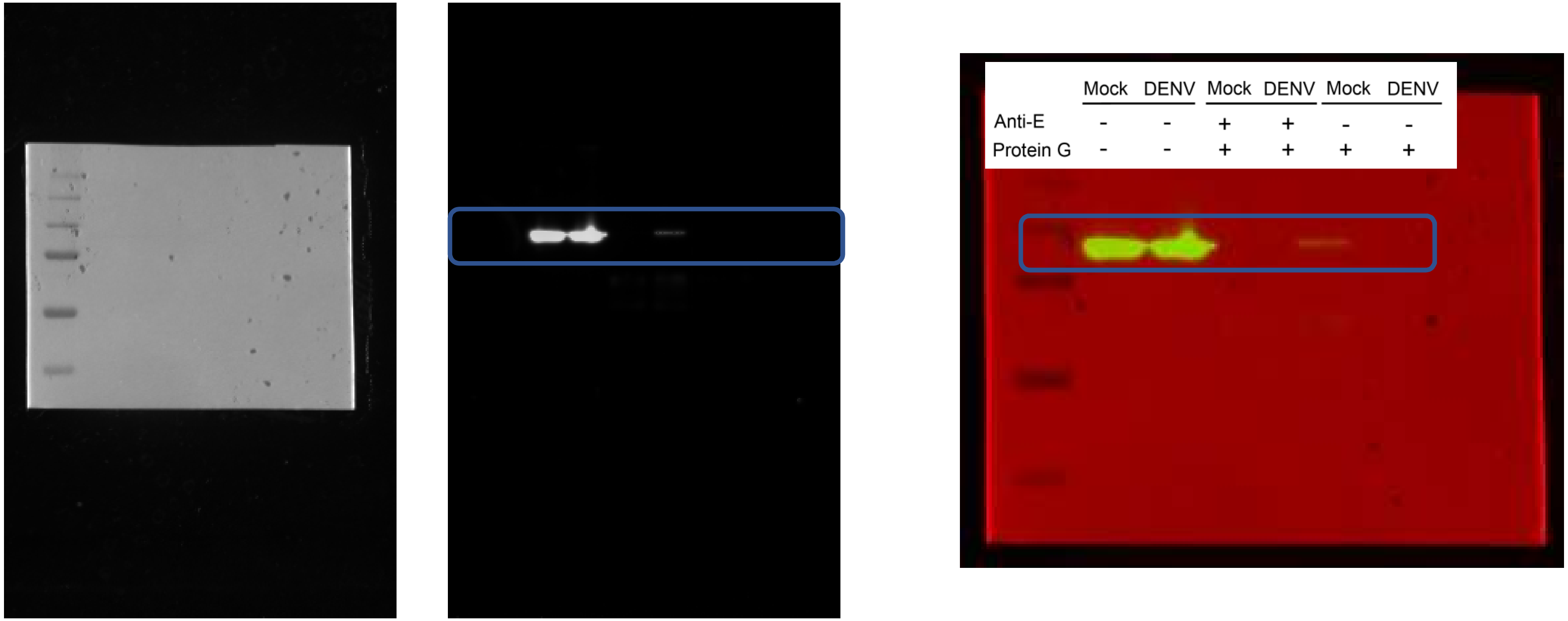

Bright field

HEK293T cell

Figure 4: IP pull down E protein

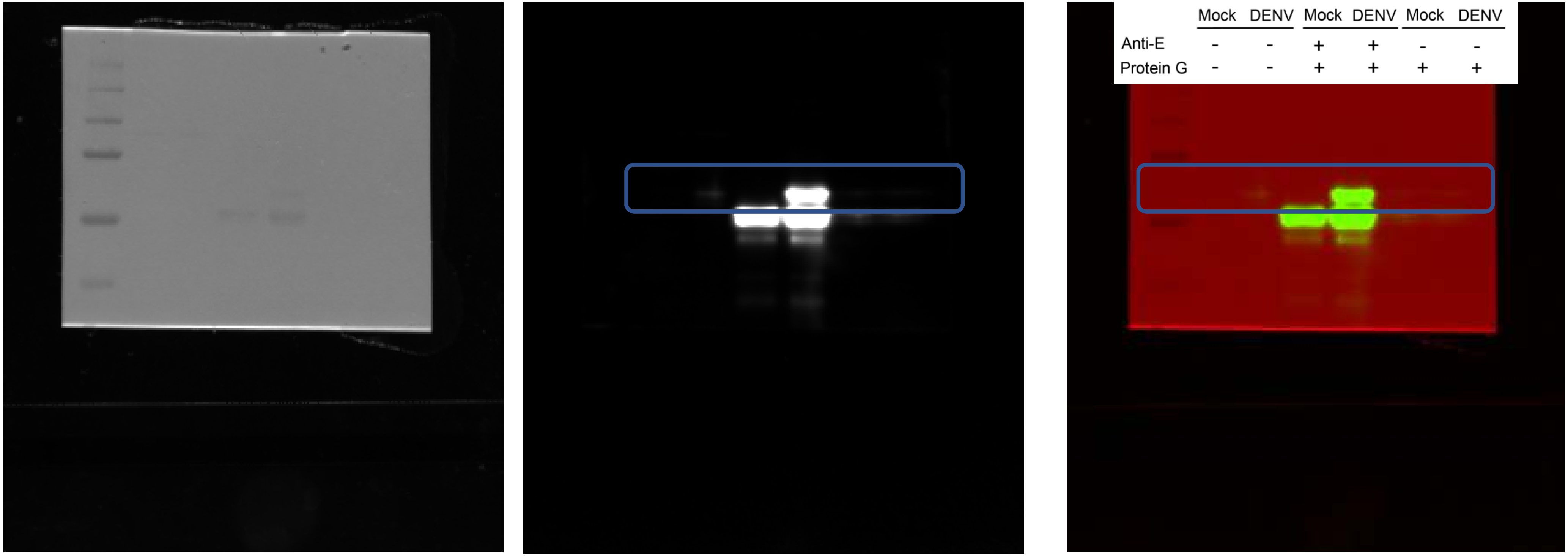

Bright field

HEK293T cell

Figure 4: reverse Co-IP pull down NS2B protein

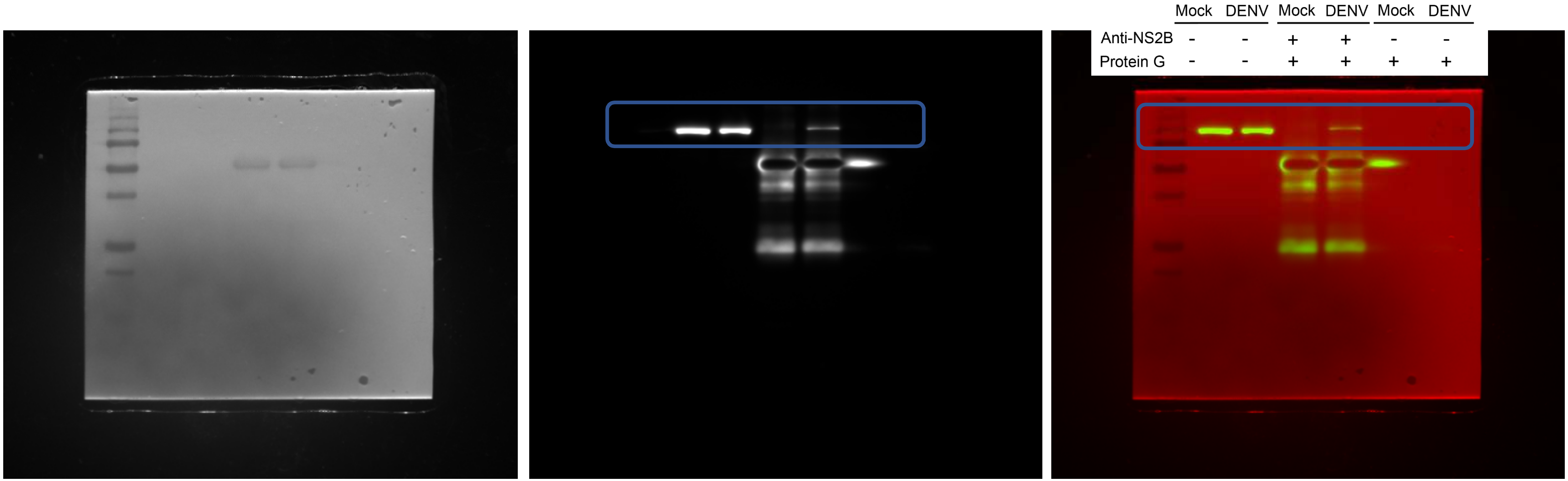

Bright field

HEK293T cell

Figure 4: IP pull down NS2B protein

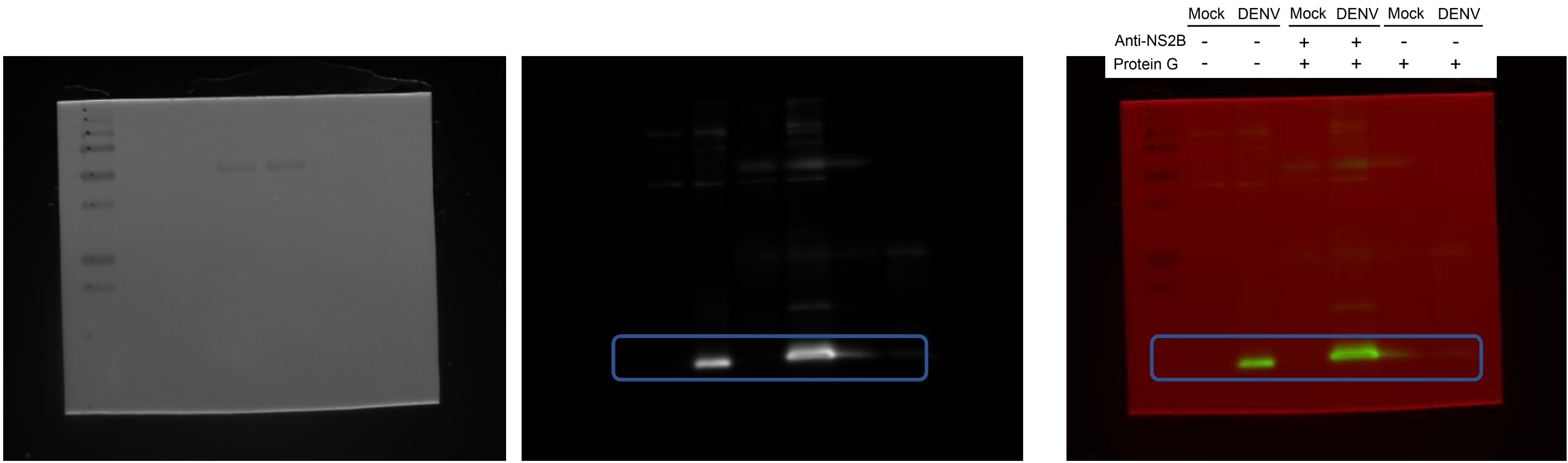

Bright field

HEK293T cell

Figure 4: reverse Co-IP pull down NS3 protein

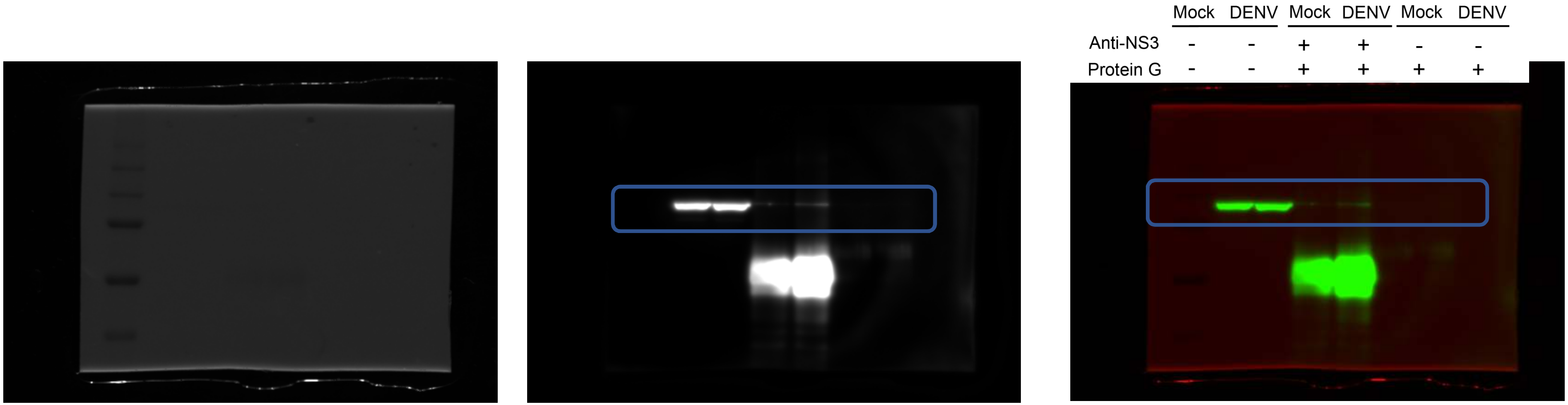

Bright field

HEK293T cell

Figure 4: IP pull down NS3 protein

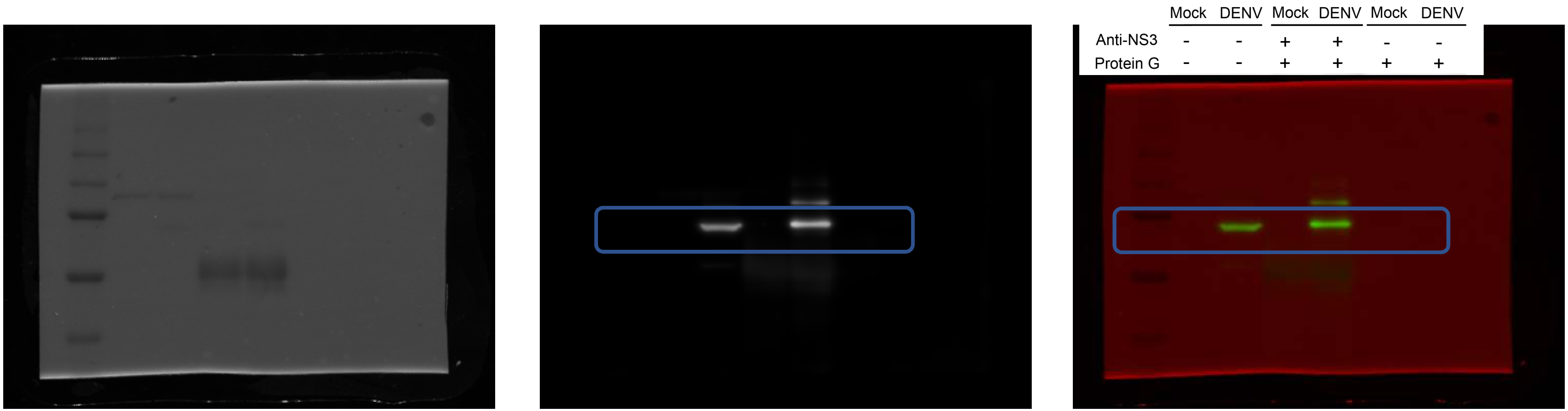

Bright field

HEK293T cell

Figure 4: reverse Co-IP pull down NS4B protein

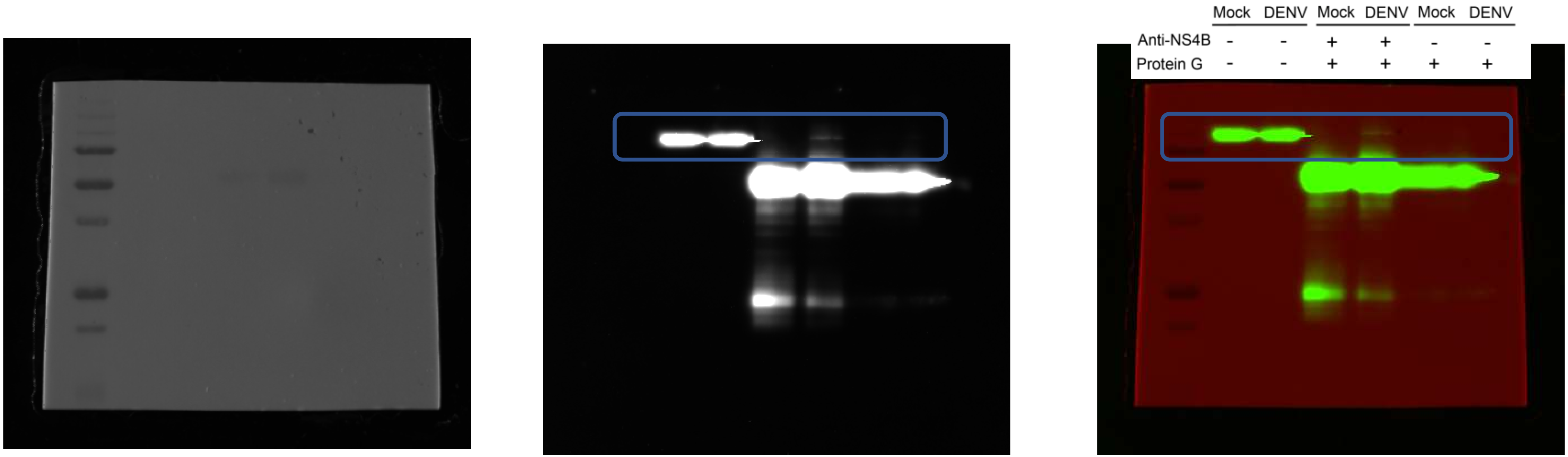

Bright field

HEK293T cell

Figure 4: IP pull down NS4B protein

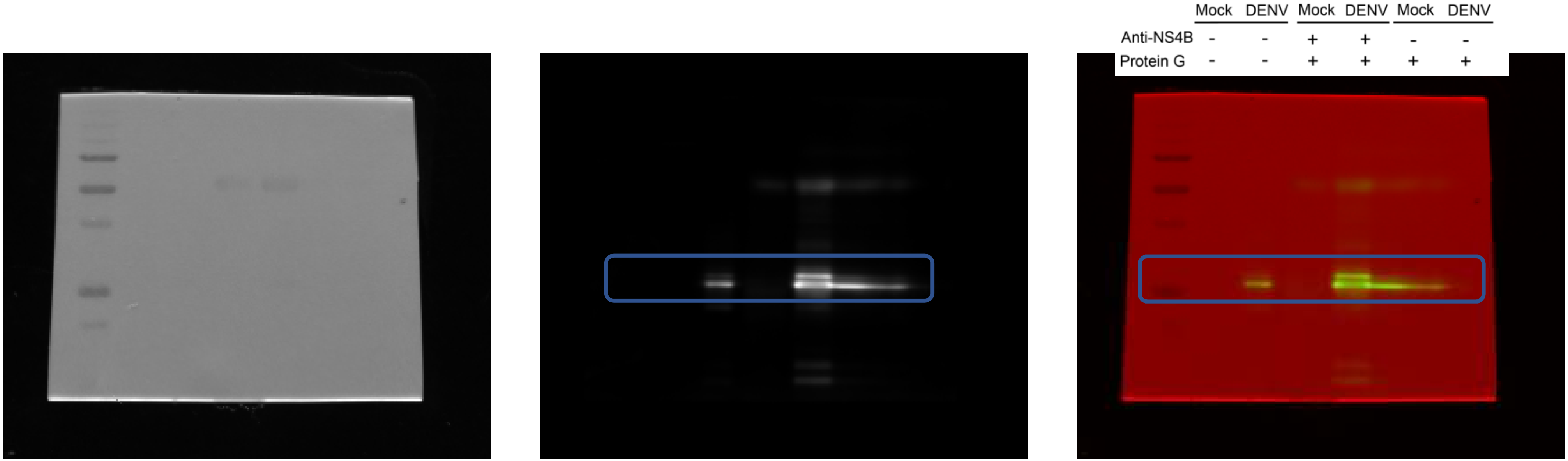

Bright field

HEK293T cell

Figure 4: reverse Co-IP pull down NS5 protein

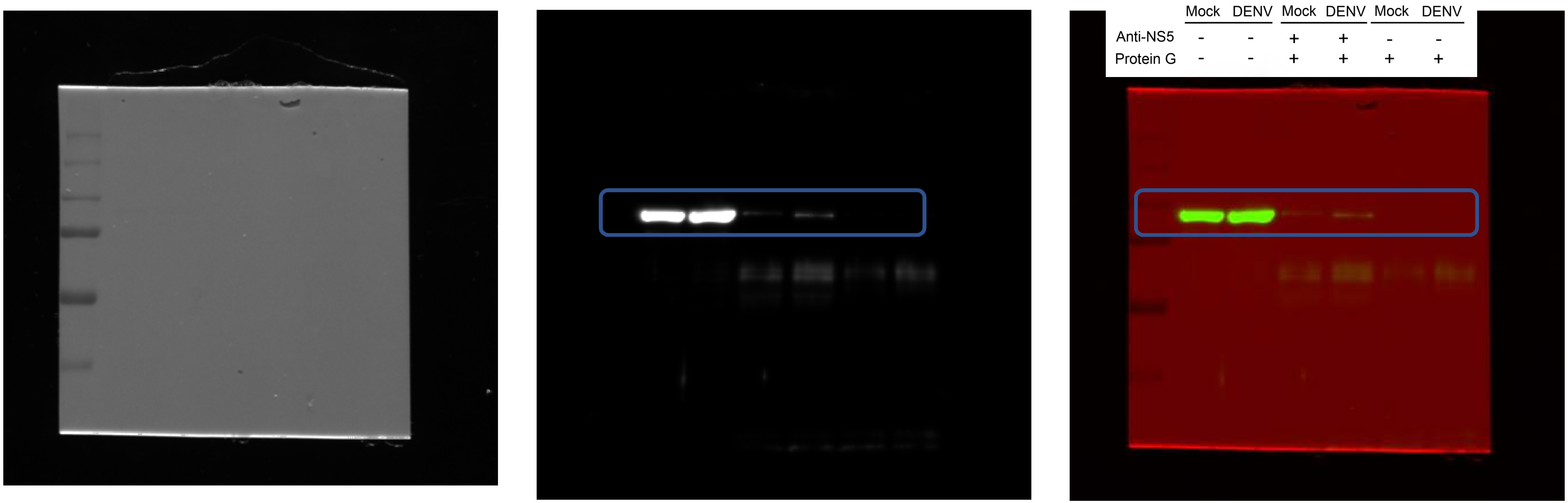

Bright field

HEK293T cell

Figure 4: IP pull down NS5 protein

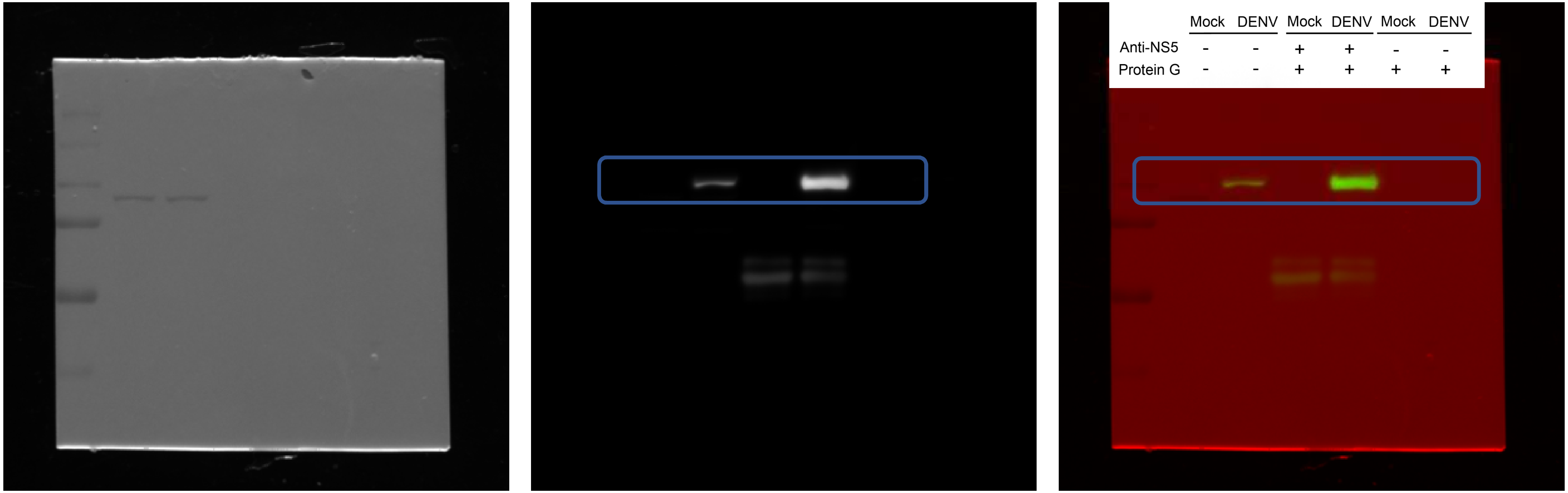

Bright field

HEK293T cell

Figure 4: reverse Co-IP pull down capsid protein

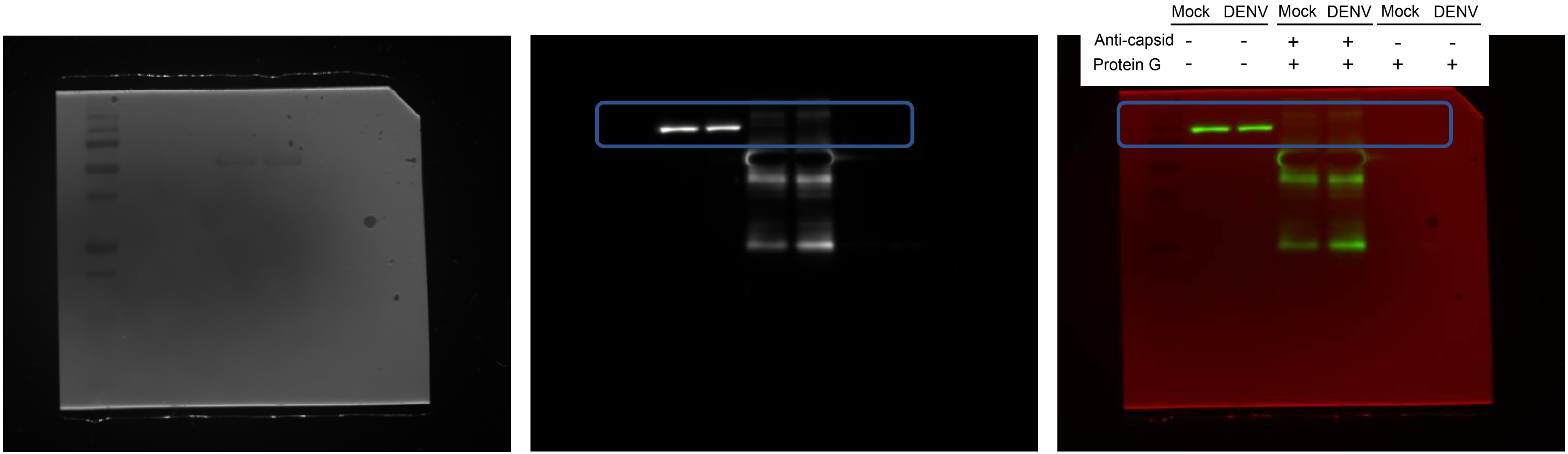

Bright field

HEK293T cell

Figure 4: IP pull down capsid protein

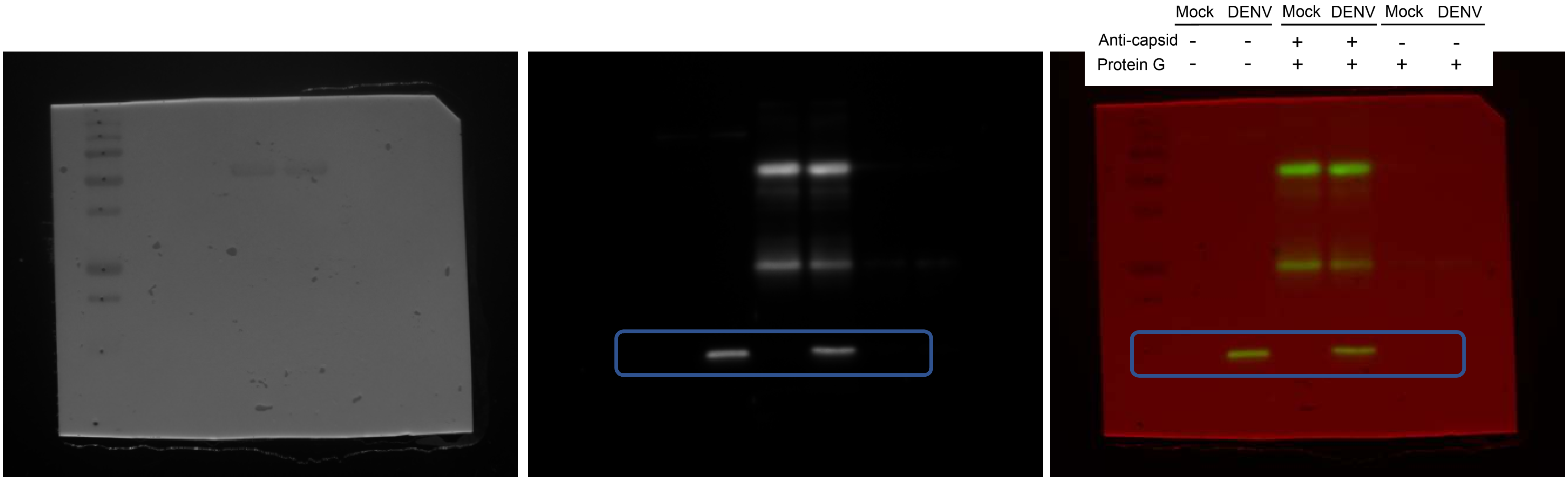

Bright field

HEK293T cell

Supplemental S1B: Check expression plasmid NS3

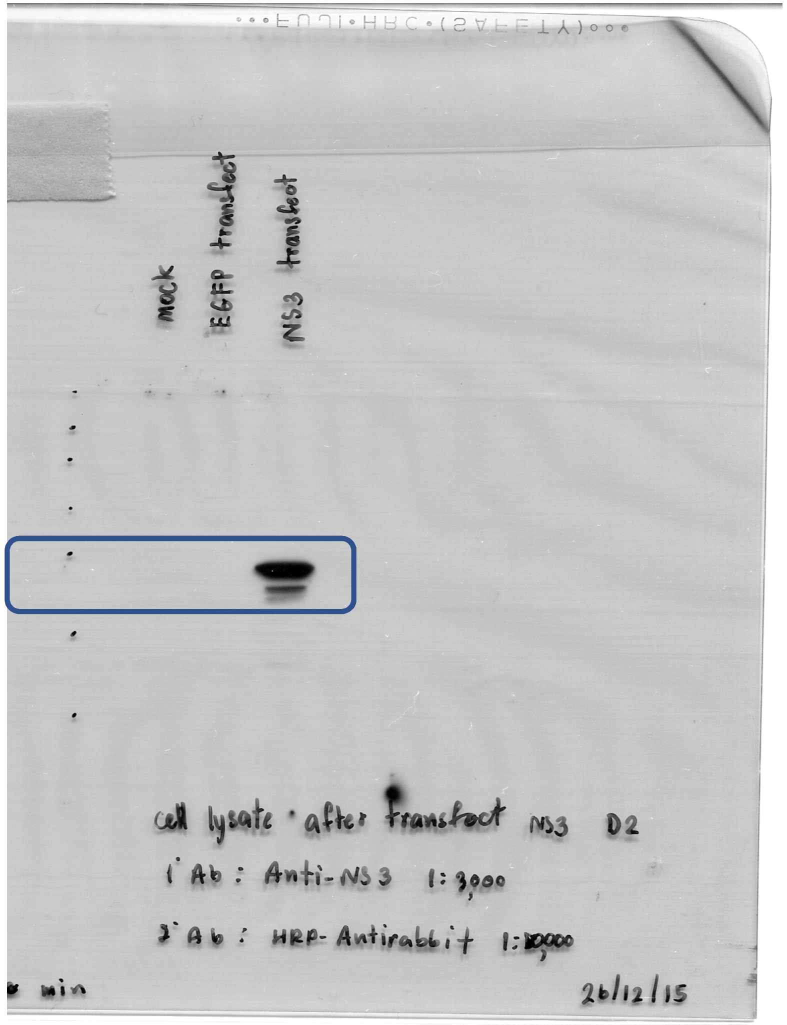

HEK293T cell

Supplemental S1B: Check expression plasmid NS5

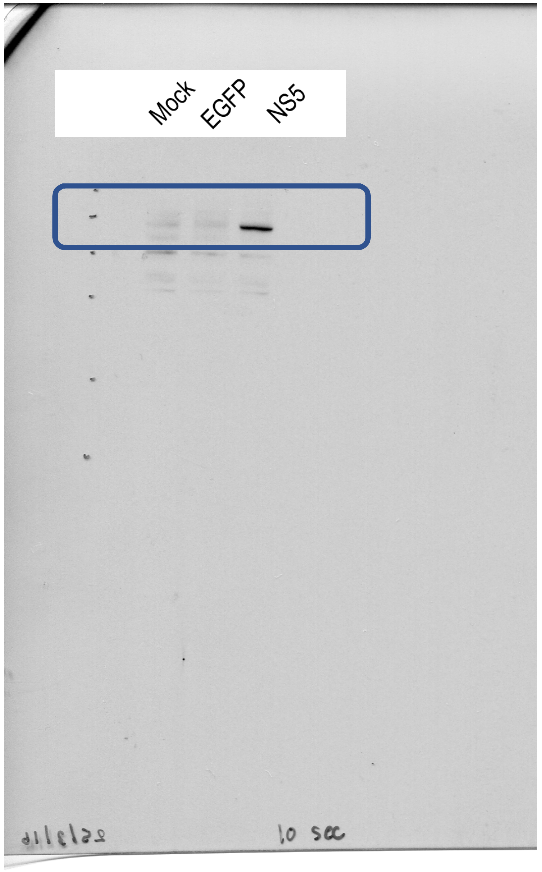

HEK293T cell

Figure 5: Co-IP transfected NS3 (probe NS3)

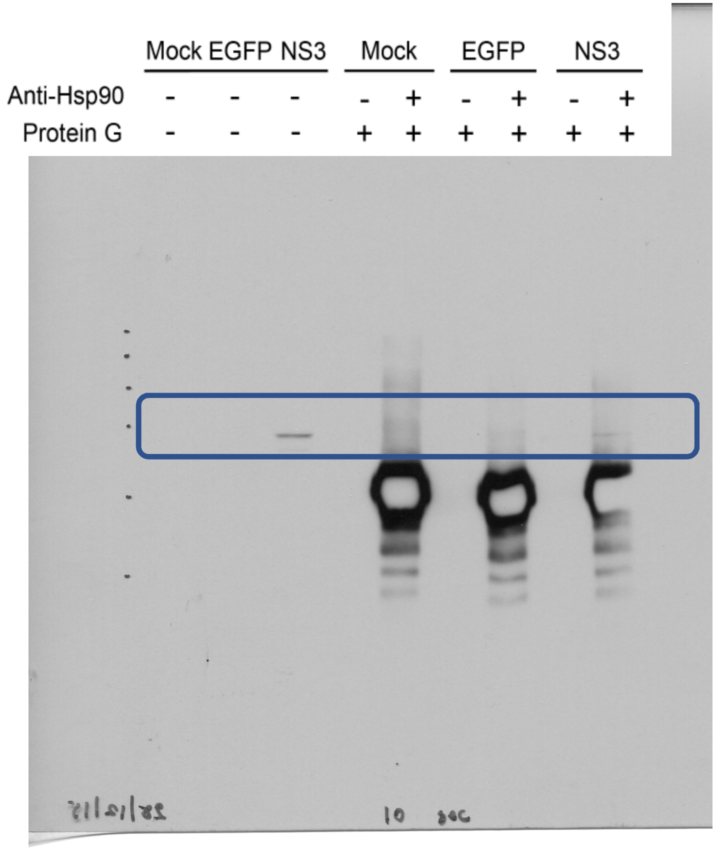

Figure 5: IP transfected NS3 (probe Hsp90)

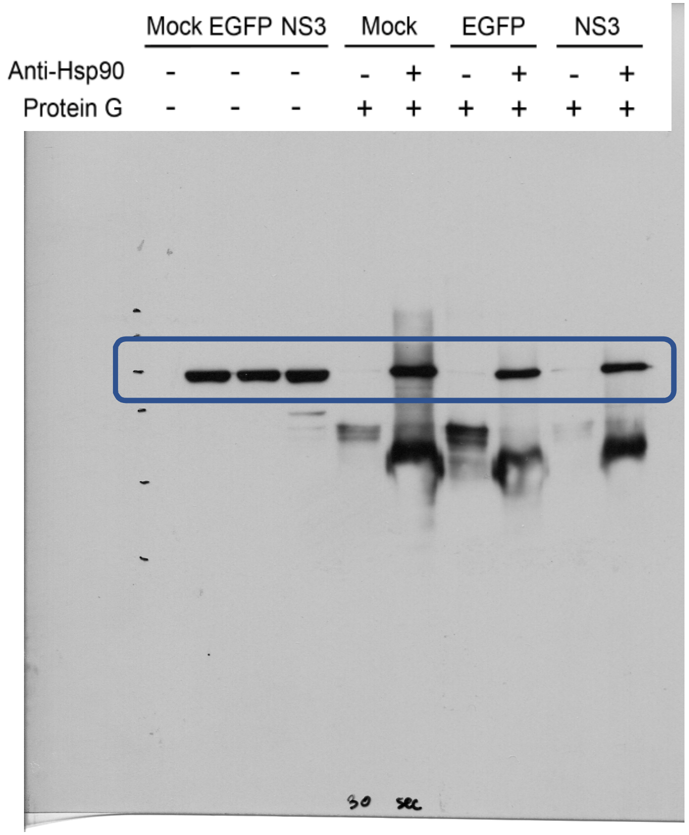

HEK293T cell

Figure 5: Co-IP transfected NS5 (probe NS5)

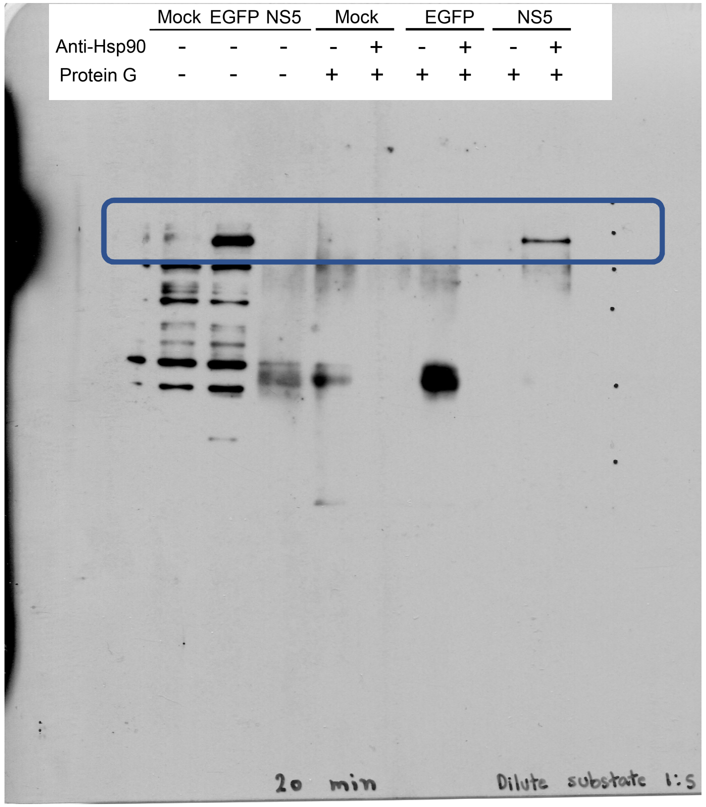

Figure 5: IP transfected NS5 (probe Hsp90)

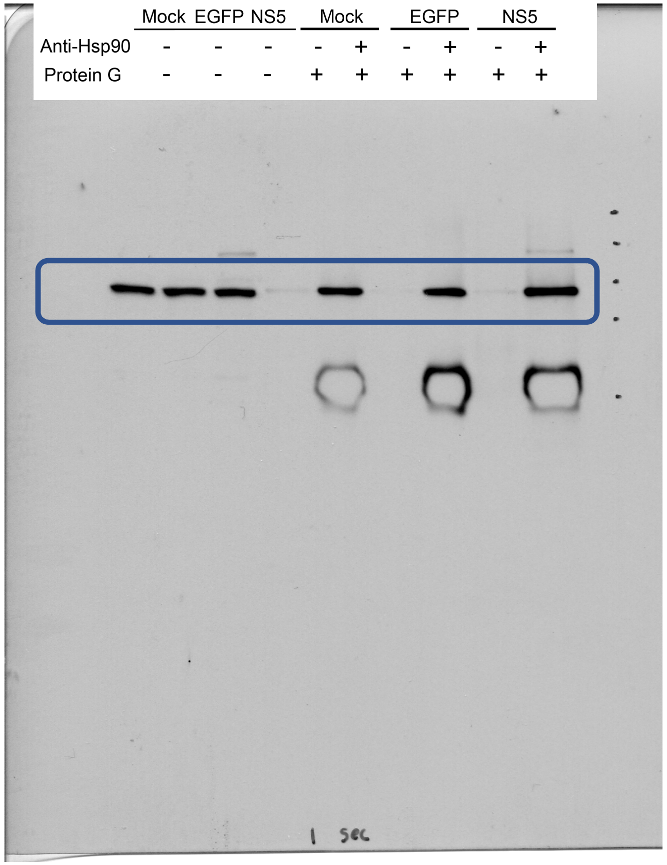

Treated HEK293T cell with GA

Figure 11C: Supernatant probe E protein

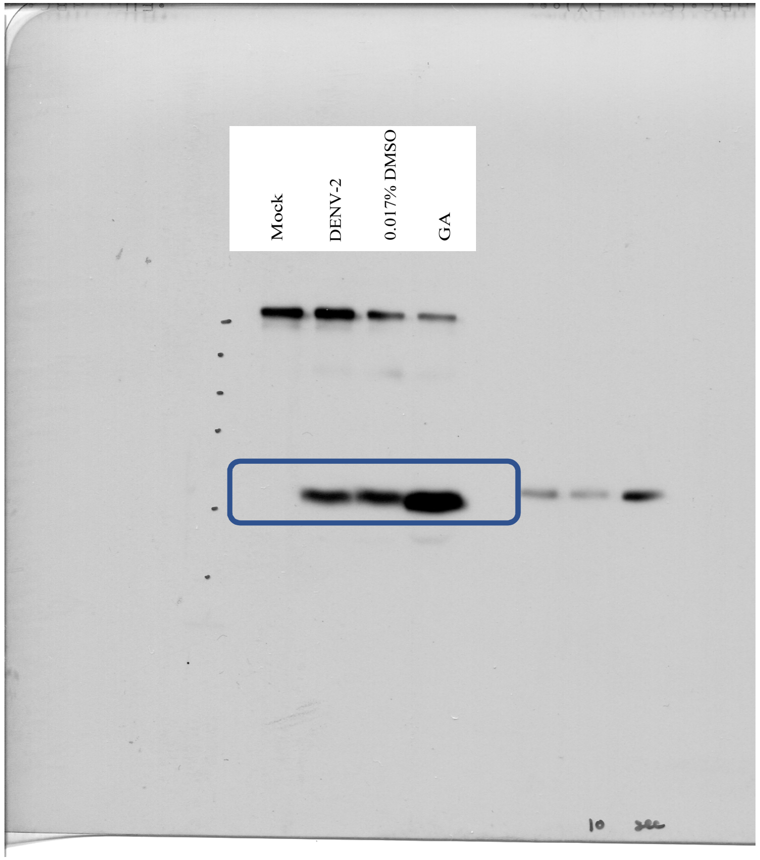

Treated HEK293T cell with GA

Figure 11D: cell lysate probe E protein

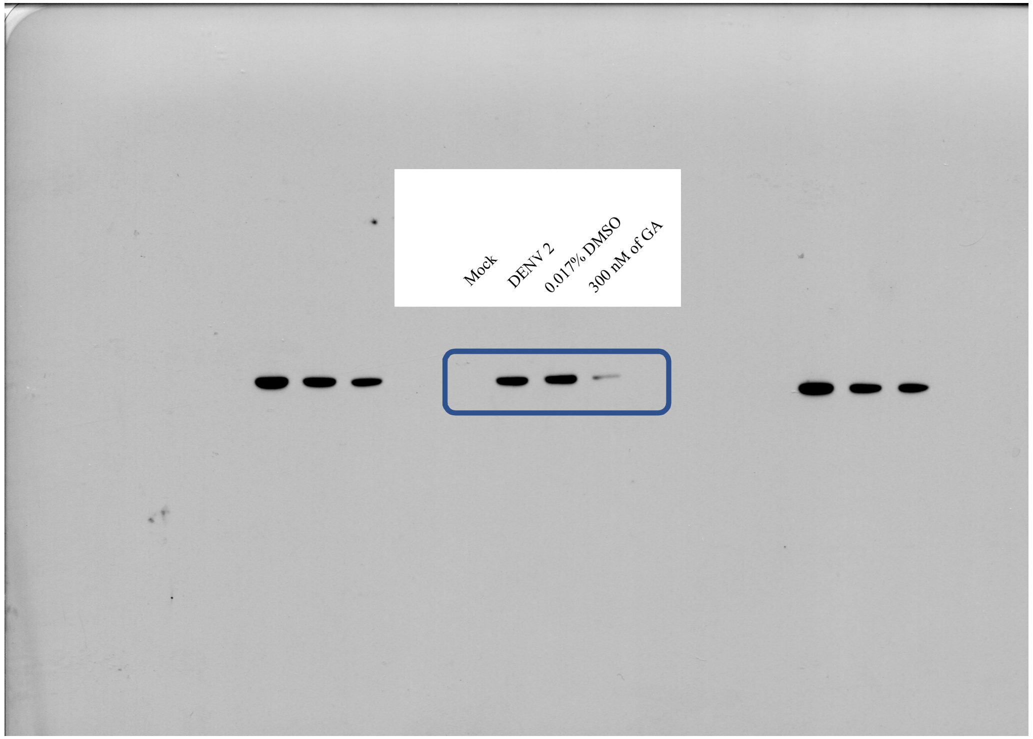

3 replicates

Figure 11D: cell lysate probe NS5 protein

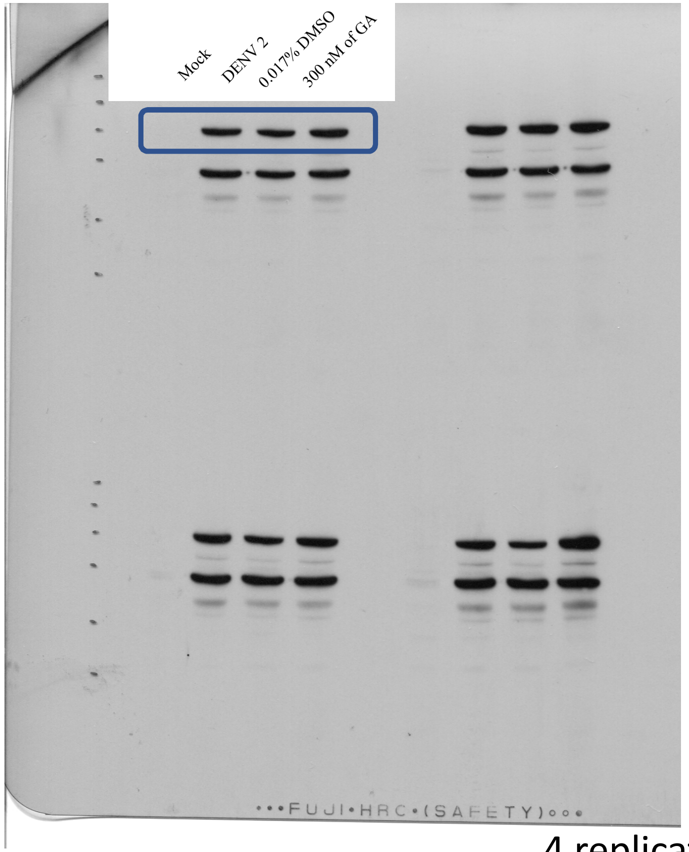

4 replicates

Treated HEK293T cell with GA

Figure 11D: cell lysate probe Hsp90 protein

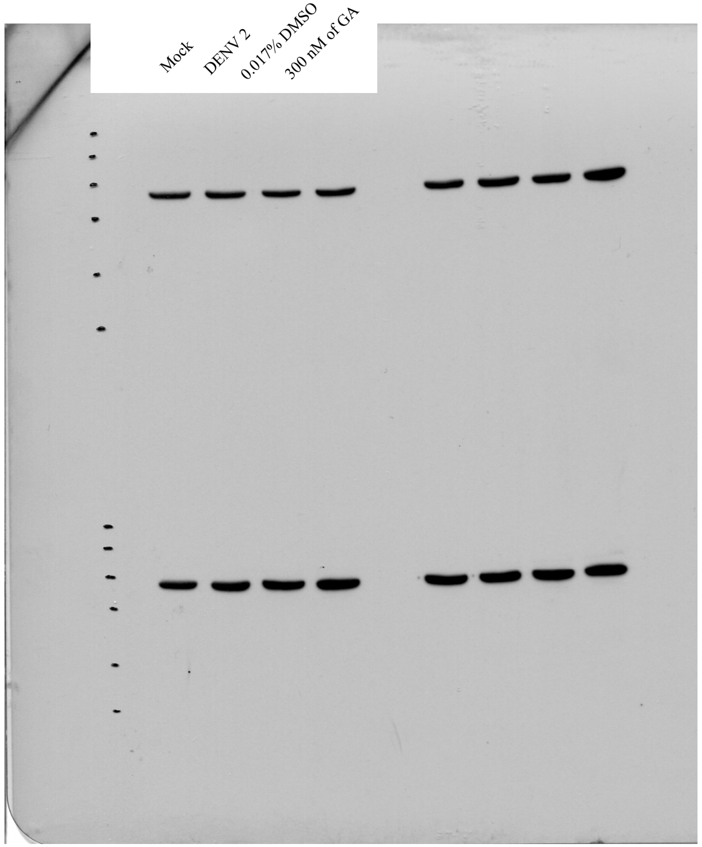

4 replicates

Figure 11D: cell lysate probe NS3 protein

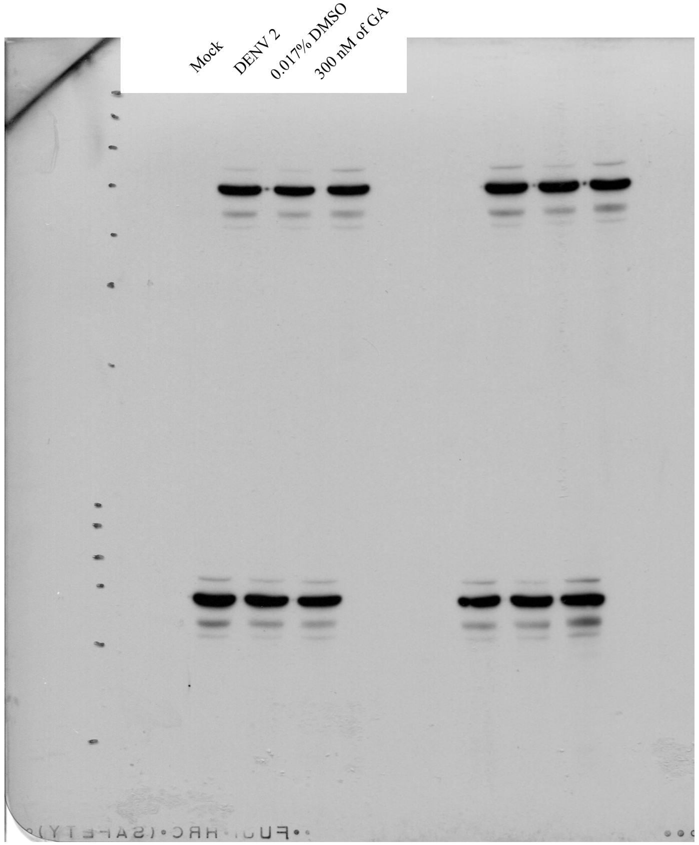

4 replicates

Treated HEK293T cell with GA

Figure 11D: cell lysate probe actin protein

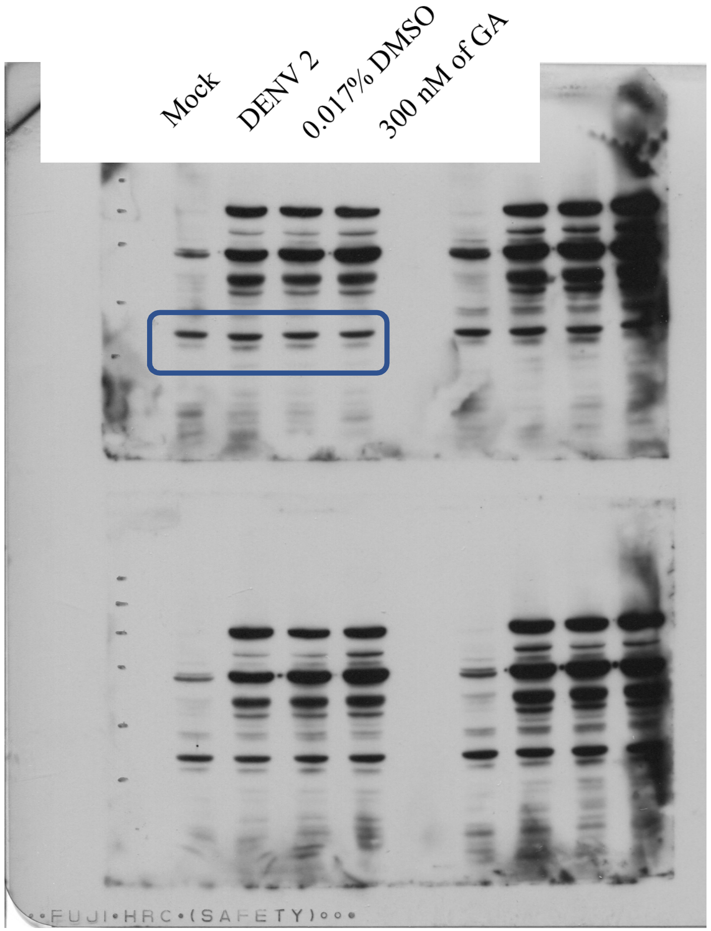

4 replicates

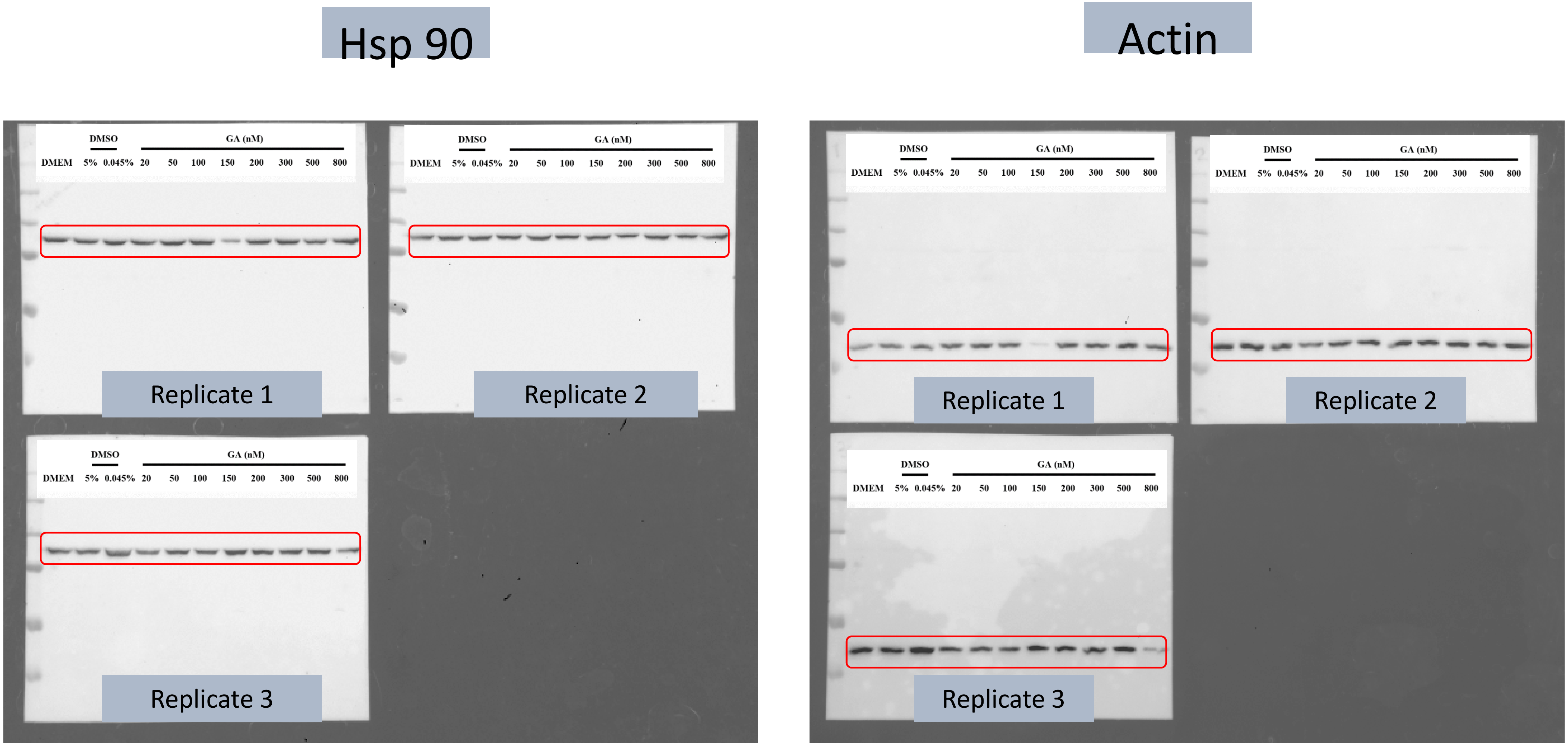

Supplemental Figure S4
